# Analysis of the spatial distribution of metabolites in *Aloe vera* leaves by mass spectrometry imaging and UHPLC-UHRMS

**DOI:** 10.1038/s41598-025-88144-8

**Published:** 2025-01-28

**Authors:** Sumi Krupa, Tomasz Ruman, Wiktoria Szuberla, Joanna Nizioł

**Affiliations:** 1 Doctoral School, Rzeszów University of Technology, 8 Powstańców Warszawy Ave., Rzeszów, 35-959 Poland; 2 Department of Inorganic and Analytical Chemistry, Faculty of Chemistry, Rzeszów University of Technology, 6 Powstańców Warszawy Ave., Rzeszów, 35-959 Poland; 3 Department of Polymers and Biopolymers, Faculty of Chemistry, Rzeszów University of Technology, 6 Powstańców Warszawy Ave., Rzeszów, 35-959 Poland

**Keywords:** *Aloe vera*, Mass spectrometry imaging, Laser ablation, Metabolomics, Plant tissue, Ambient ionization, Analytical chemistry, Plant physiology

## Abstract

This study presents an investigation of the chemical composition of *Aloe vera* leaf tissue with a focus on the spatial distribution of compounds. The composition was studied using two mass spectrometry imaging techniques: silver-109 nanoparticles assisted laser desorption/ionization mass spectrometry imaging (^109^AgNPs-LDI-MSI) and laser ablation-remote atmospheric pressure photoionization/chemical ionization mass spectrometry imaging (LARAPPI/CI-MSI) and the identification was aided by ultra-high-performance liquid chromatography and ultra-high-resolution mass spectrometry (UHPLC-UHRMS) analysis. The results showed an abundance of phenolic compounds with antioxidant, antimicrobial, and anti-inflammatory properties, making it a beneficial food additive and food packaging material. Analysis of the results of mass spectrometry imaging provided information about the potential changes in metabolic pathway expression in different regions of the leaf.

**Supplementary Information:**

The online version contains supplementary material available at 10.1038/s41598-025-88144-8.

## Introduction

According to market research^1^ the value of the global *Aloe vera* market in 2023 was 2.65 billion dollars and is projected to grow to 2.86 billion USD in 2024. The market share connected to using *A. vera* in the food and beverage industry is evaluated at approx. 25% of the global market value. Aloe has been utilized in the human diet in different forms, starting with the consumption of raw parts of the plant. The flowers of *A. vera* have been regarded as edible and their nutritional contents have been studied, revealing the occurrence of β-sitosterol, β-carotene, and tocopherol, as well as lipids, such as palmitic acid^2^. The products made with added aloe extracts include drinks, such as juices or health-promoting beverages^3,4^, products of milk fermentation, jams, and jellies^5,6^. *A. vera* gel has been found useful as an edible coating for increasing the shelf-life of perishable foods^7–9^.

Due to its properties, *A. vera* has been utilized in other fields. It has found use in cosmetics thanks to the presence of polysaccharides, which stimulate fibroblasts, effectively preventing skin irritation, likewise protecting from UV-B-induced hazards^10^. Oligosaccharides present in *A. vera* gel also exhibit an effect in reduced Interleukin-10 production, which results in UV-induced skin hypersensitivity prevention^11^. This, coupled with the moisturizing, soothing, antibacterial, and anti-inflammatory action of *A. vera* gel resulted in its common use as an ingredient in creams, cleansers, and moisturizers^12^. A wide set of applications for *A. vera* is documented for its medicinal qualities. Mannose-6-phosphate^13^, aloesin, emodin, and aloin^14–16^ present in *A. vera* are associated with the reduction of inflammation and antioxidant mechanisms, aiding in wound healing. *A. vera* gel used on the skin can also result in glycosaminoglycans and type III collagen production, further promoting tissue healing^17^. *A. vera* can be used in HSV-1 treatment^18^, as well as psoriasis^19^ and seborrheic dermatitis^20^. Aloe exhibited antidiabetic potential connected to its antioxidant qualities^21^.

The analytical methods most commonly used for *A. vera* analysis include chromatography coupled with mass spectrometry, such as gas chromatography (GC-MS)^22^ and liquid chromatography (LC-MS)^23^, as well as nuclear magnetic resonance spectroscopy (NMR)^24^. Those methods, however, require that the extract of the homogenized sample is used, which results in a loss of information about the location of identified compounds. Mass spectrometry imaging (MSI) allows for the detection of various compounds in the analyzed sample, usually a tissue, without previous homogenization, resulting in the acquisition of data in the form of ion images with the spatial location of each compound in the sample.

MSI has been used in plant tissue analyses investigating plant growth and reproduction processes^25^. The metabolic processes in endosperm have been studied, as well as the effect of light availability to specific parts of a leaf on secondary metabolite accumulation. Investigation into the nitrogen cycle and the effect of symbiotic microorganisms in the process has also been conducted. MSI also allowed for the analysis of plant biotic and abiotic stress responses^26^. Due to the possibility of visual comparison of the regions where the compound occurs in the sample in the highest amount, a better understanding of the metabolism and functions of different parts of the organism is possible. The analysis of plant tissues can prove problematic due to the difference in anatomy between different structures of the plant organs. *A. vera* leaf is made up of three differentiable regions. The structured outermost layer is responsible for the transportation of substances due to numerous vascular bundles, a middle layer, close to the epidermis, is made of sap containing glycosides and anthraquinones, and the inner layer – gel – is composed of up to 98% water with various compounds mostly sterols, polyphenols, and alkaloids^27^. The preparation of tissue with highly varying structural integrity regions poses technical difficulties due to differences in sample material desorption, as well as in the ionization of compounds, especially in the gel region where the low concentration of compounds other than water makes it difficult to detect or image certain molecules.

Various techniques provide the possibility of desorption and ionization in MSI analysis, so it’s important to choose the method most suitable for the desired results and sample type. The most common techniques are desorption electrospray ionization (DESI), secondary ion mass spectrometry (SIMS), and laser desorption/ionization methods, most notably matrix-assisted laser desorption/ionization (MALDI).

MSI analysis conducted using SIMS produces ion images with the highest resolution of all mentioned MSI techniques^28,29^. This in turn is connected to imaging of a smaller size of the sample region. In this study, the whole cross-section of a leaf was analyzed, which wouldn’t be possible for SIMS. The method is also regarded as a hard ionizing one, meaning that fragments of precursor ions are produced alongside molecular ions. For the analysis of complicated biological systems with a variety of compounds, soft ionization methods are preferred.

MALDI is the most common ionization technique for plant tissue MSI. MALDI utilizes an organic matrix as surface assistance^30^. The use of organic matrices interferes with the signals on the spectrum in the lower mass region (< 1000 Da), which is why the use of inorganic matrices, such as nanoparticles (NPs) in surface assisted-laser desorption/ionization (SALDI) became very popular. A considerable problem is the presence of “sweet spots” occurring when conducting a MALDI-MSI analysis. The solution again lies in the use of nanoparticles, as they do not exhibit this effect. Ions emitted from nanoparticles can also serve as internal calibration points^31^. The process of sample preparation for MALDI-MSI is time-consuming and complicated, as the tissue has to be sliced to the width of ~ 10 μm and homogeneously covered with the used matrix.

Both SIMS imaging and LDI-MSI analyses are conducted in vacuum conditions. Biological samples can change their shape in those conditions due to the loss of moisture. The imprinting method can be used to overcome that, as no actual tissue enters the high vacuum conditions^32,33^, however, the material that is transferred represents mainly intercellular liquid. DESI-MSI has the advantage of analysis in ambient conditions in contrast to the aforementioned methods, which contributes to sample integrity conservation, however, the resolution achievable in DESI-MSI analysis is the lowest out of the mentioned techniques and the sampling depth is very shallow^34^.

Biological samples contain a substantial amount of water, which makes a mid-IR laser perfect for the desorption of the material from the sample for ionization. Mid-IR wavelength, specifically 2930 nm in the case of this study, effectively couples its energy into the stretching mode of the O-H bond, which makes it very effective for the desorption of highly hydrated biological material, such as *A. vera* leaves. Laser ablation-remote atmospheric pressure photoionization/chemical ionization (LARAPPI/CI) that uses this laser is a novel technique used in mass spectrometry, developed by Ruman et al.^35^. This technique allows for MSI analysis of tissues in a close-to-native state after freezing a sample without any previous preparation. The superficial resolution of the technique is 140 μm and the depth of sampling is from 100 to 300 μm depending on the sample and pulse energy. LARAPPI/CI system is capable of 2D as well as 3D imaging.

In this study, two very different MSI methods: 109-silver nanoparticles assisted laser desorption/ionization mass spectrometry imaging (^109^AgNPs-LDI-MSI) and LARAPPI/CI-MSI were used to determine the *A. vera* leaf location of metabolites. The identification was aided by UHPLC-UHRMS + MS/MS analysis of the extract from the plant tissue. This is the first work that contains the comparison of nanoparticles-based laser mass spectrometry imaging with chemical ionization/photoionization-based imaging of plant tissue.

## Experimental

### Materials

A silver-109 isotope of 99.7% isotopic purity was bought from Trace Sciences International (USA). All solvents (toluene, chloroform, acetonitrile) were of HPLC quality, except for water (18 MΩcm water produced locally). The plant used in this study was initially purchased from a local store in Rzeszow, Poland. The analyzed samples for MSI analyses were cross-section slices of *A. vera* (L.) leaf obtained from a 5-year-old potted plant, which had been cultivated under indoor conditions in southern Poland for two years following its purchase. Half of a leaf, growing at the midpoint of the trunk, was cut off from the stem. The age of the analyzed leaf fragment is estimated to be 2 years (± 3 months). A leaf was collected from the middle section of the plant and transported at 4 °C. It was analyzed within 10 min of collection, without further storage in the laboratory. Prior to analysis, the *A. vera* leaf was cut at one-third of its length from the tip. For ^109^AgNPsLDI-MSI, the entire cut section was used to create a cross-sectional imprint on the steel plate (LDI target plate). For LARAPPI/CI-MSI, a 3.75 mm thick slice was prepared by cutting with a blade from the region of the leaf adjacent to the one used for imprinting (for LDI-MSI), then placed on the stainless steel plate and frozen. For LC-MS analysis, 200 mg of *A. vera* material from the leaf region adjacent to that used for MSI analyses was used.

Steel plates used for LARAPPI/CI-MSI as well as LDI targets were fabricated from H17 stainless steel and underwent a thorough cleaning process prior to MSI. The cleaning involved sequential soaking in boiling solvents, including toluene, chloroform, acetonitrile, and deionized water (each solvent was used three times in 100 mL volumes for 30 s per plate). After cleaning, the plates were dried under high vacuum conditions (approximately 0.01 mbar) for 24 h. Optical photographs were made with the use of an Olympus SZ10 microscope equipped with an 8 MPix. Olympus digital camera and also a Canon 6D camera with a macro-type 90 mm focal length lens.

### Sample extraction for LC‑MS analysis

*A. vera* leaf material was homogenized with 1050 µL of 3:1 acetone/water (v/v) mixture in a bead homogenizer. After homogenization, the samples were placed at a temperature of -20 °C for 20 min. After this time, the samples were centrifuged for 10 min at 9800 x g. The supernatant was then transported to Eppendorf vials and dried in a SpeedVac vacuum concentrator for 18 h. The samples were weighted after drying and 100 µL of methanol was added per every 1 mg of the dried extract. Samples were then centrifuged again in 9800 x g for 5 min, 100 µL from each vial was placed in HPLC vial inserts and inserted into a Bruker Elute autosampler. The samples were in a thermostated autosampler at 4 °C during the analysis.

### Imaging of *A. vera* leaf cross-section imprint using ^109^AgNPs-LDI-MSI

Imaging of the cross-section imprint of the *A. vera* leaf was performed following the methodology described in our previous publications^36^. Fresh leaf was cut transversely at one-third of the distance from the tip, and excess liquid from the cut surface was absorbed using filter paper. The leaves were manually imprinted onto the surface of the steel target plate by gently pressing for 3 s. Next, the leaf was removed, and the remaining imprint on the plate was coated with a suspension of silver-109 nanoparticles using the pulsed fiber laser-generated nanomaterial method with a 2D galvo-scanner (PFL 2D GS LGN)^37^. The process of the 109-silver nanoparticles synthesis and application on the surface of the plate was described in our recent publication^38^. Measurements were performed using a Bruker Autoflex Speed time-of-flight mass spectrometer in reflectron positive ion mode. The apparatus was equipped with a Smart Beam II 1000 Hz 352 nm laser. Laser impulse energy was approximately 100–190 µJ, laser repetition rate was 1 kHz, and deflection was set on *m*/*z* lower than 95. The measured *m*/*z* range was 100–2000, experiments were made with 2000 laser shots per individual spot. All spectra were calibrated with the use of silver ions of ^109^Ag^+^ to ^109^Ag_13_^+^ formula.

### LARAPPI/CI-MSI of *A. vera* leaf cross-section

The MSI in ambient conditions was performed using the LARAPPI/CI technique described in our recent publication^35^. For LARAPPI/CI-MSI analysis, a 3.75 mm thick slice from the same region of the *A. vera* leaf was placed on a clean stainless steel plate. The plate was then mounted on the stage with a Peltier cooling plate (TE-127-1.4-1.5; TE Technology, Traverse City, MI, USA) which was set to -18 °C. This ensured the sample remained frozen throughout the laser ablation process, minimizing the risk of compound mixing on the sample surface. An Nd/YAG-pumped, tunable OPO laser (Opolette HE 2940; Opotek, Carlsbad, CA, USA) was used to generate mid-infrared (mid-IR) laser pulses. The laser system produced shorter than 7 ns pulses with a maximum repetition frequency of 20 Hz. The laser was tuned to emit at a wavelength of 2.93 μm. The pulse energy of the laser-measured before focusing lens was 2.0 mJ. The process of laser ablation was conducted in an airtight chamber pressurized with nitrogen gas at a steady flow of 9.5 L/min. The mass spectrometer used for the analysis was Bruker Impact II operating in negative ion mode. The ionization was conducted using Bruker VIP HESI ion source in the APCI configuration. The ion source additionally had a VUV source (Hamamatsu L12542) mounted to the MS sampling cone to induce photoionization. A binary HPLC pump (Agilent G1312A) was used to provide a steady flow of a solvent mixture (1% toluene in methanol; 200 µL/min) to the APCI needle. The laser ablation plumes were transported to the ion source through a PTFE tube connected to the ablation module. The analyzed region encompassed the whole leaf slice. The selected area was irregular in shape, fitting the shape of the slice. The spatial resolution chosen for the analysis was 200 μm. The duration of each laser shot was 1 s. To ensure accurate alignment of each pixel, the delay between the shots in one line was 1 s, and between sequential lines, it was 5 s. The analysis of the results of the imaging was done using software made specifically for the used system.

### UHPLC-Q-ToF-UHRMS analysis

UHPLC-Q-ToF-UHRMS analysis was conducted with Bruker Elute UHPLC system, coupled with a Bruker Impact II mass spectrometer of ESI QToF-MS type. The UHPLC column used was the C18 Bruker Intensity Solo with silica functionalized with octadecyl groups, with 2 μm particles and dimensions of 100 × 2.1 mm (length × diameter). To maintain consistent conditions, the UHPLC column was thermostated at 40 °C during the analysis. For mobile phases, water with 0.1% HCOOH as phase A and acetonitrile with 0.1% HCOOH as phase B were used. The injection volume was set at 5 µL, and the percentage of phase B was as follows: 1% (0–2 min), 99% (17–20 min), and 1% (20.1–30 min). From 0 to 20 min, the solvent flow rate was 0.25 mL/min, gradually increasing to 0.35 mL/min from 20.1 to 30 min. All measurements were made in technical triplicates. Measurements in positive autoMSMS mode were carried out using the following parameters: *m/z* 50–1200; capillary voltage: 4.5 kV; nebulizer: 2.7 bar; dry gas: 12 L min^-1^; drying gas temperature: 220 ^o^C; hexapole voltage: 50 Vpp; funnel 1: 200 Vpp; funnel 2: 200 Vpp; pre-pulse storage time: 5 µs; transfer time: 60 µs. Collision-induced dissociation (CID) was used with the following settings: absolute threshold (per 100 sum): 200 cts; absolute threshold 88 cts; active exclusion 3 spectra; release after 0.3 min, isolation mass: for *m/z* = 100, the width was 3, for 500 widths was 4, for 1000 was 6 and for 1300 was 8); collision energy value was 30 eV. MS frequency was 20 Hz and for MS/MS - from 5 to 30. The untargeted annotations were performed in Metaboscape (ver. 2022b) with a criterion of mass deviation (Δ*m/z*) under 2 ppm and mSigma value under 20 as the maximum acceptable deviation of the mass of the compound and the isotopic pattern respectively. For identification and molecular formula generation, the exact mass of parent ions was matched with < 3 ppm error and mSigma value < 50 in most cases. All the molecular formulas were obtained using the Smart Formula tool and the C, H, N, O, P, S, Cl, Br, I, and F elements. MS/MS spectra were automatically matched against MS/MS libraries: Bruker HMDB 2.0 (this database contains retention times that were used as an additional identification factor), MassBank of North America (MoNA) library, and NIST ver. 2020 MS/MS library. The matching of identified compounds with metabolic pathways of the *Arabidopsis thaliana* plant was conducted based on the Kyoto Encyclopedia of Genes and Genomes (KEGG) database. The analysis of metabolic pathways in *A. vera* leaf was conducted in MetaboAnalyst 6.0 software^39^.

## Results and discussion

The analysis of the composition of *A. vera* leaf was conducted by three analytical methods. The mass spectrometry imaging analyses of the *A. vera* leaf cross section were conducted by two methods: ^109^AgNPs-LDI-MSI and LARAPPI/CI-MSI. The identification process was initiated through the analysis of *A. vera* leaf extracts using UHPLC-UHRMS and MS/MS detailed in Sect. 2.5. The additional MSI images were also generated for compounds reported for *Aloe* genus plants in published analytical studies. Based on the identified and reported compounds, ion images for exact *m/z* values were generated in MSI software using both methods (^109^AgNPs-LDI-MSI and LARAPPI/CI-MSI), taking into account various possible adducts. The analyses were performed using high-resolution instruments equipped with a ToF analyzer, which facilitated the accurate determination of molecular masses. Mass-to-charge values as well as isotopic patterns were checked for ions identified in MSI studies of both methods. The full results of the MSI analysis are presented in Fig. S1–S10 and in Table S2 in the Supplementary Information. A detailed description of UHPLC-UHRMS findings is provided in Sect. 3.2, as well as in the Supplementary Information in Table S1 and Fig. S11.

### Mass spectrometry imaging results of *A. vera* leaf

**Fig. 1:**
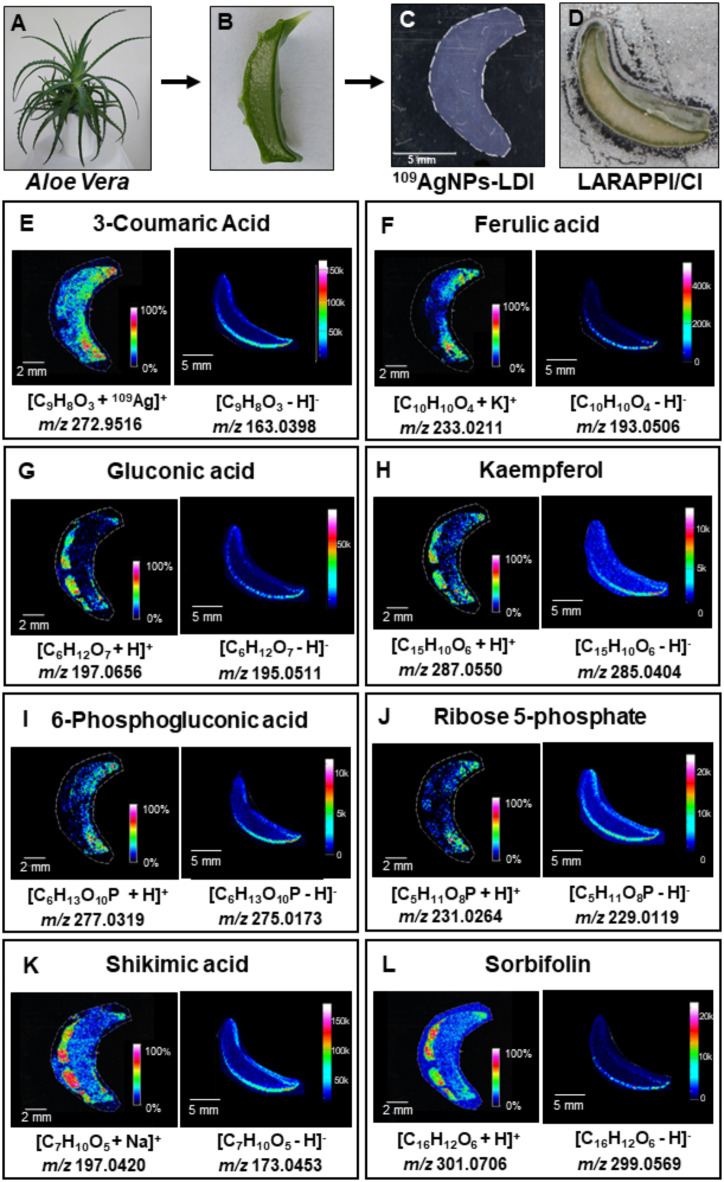
Metabolites identified by LC-MS, LDI-MSI and LARAPPI/CI-MSI. The photos taken of the sample material: (**A**) The *A. vera* plant used to acquire samples for the study, (**B**) *A. vera* leaf slice used as the sample, (**C**) the image of the imprint used for the ^109^AgNPs-LDI-MSI analysis, (**D**) the image of leaf slice used in the LARAPPI/CI-MSI analysis. The compounds identified by the three mentioned analytical methods: (**E**) 3-coumaric acid, (**F**) ferulic acid, (**G**) gluconic acid, (**H**) kaempferol, (**I**) 6-phosphogluconic acid, (**J**) ribose 5-phosphate, (**K**) shikimic acid, (**L**) sorbifolin. For each compound, the left-ion image is for the imprint technique and the right-ion image for ablation.

In ^109^AgNPs-LDI-MSI, an imprint of the *A. vera* leaf cross section was analysed. The analysis in total led to obtaining 179 ion images of identified compounds (Fig. 1C). Taking into consideration that multiple adduct types are possible, the number of identified compounds was reduced to 129. For LARAPPI/CI-MSI the number of ion images matching the sample (Fig. 1D) obtained was 122. In this method, only [M-H]^−^ ions were considered.

**Fig. 2:**
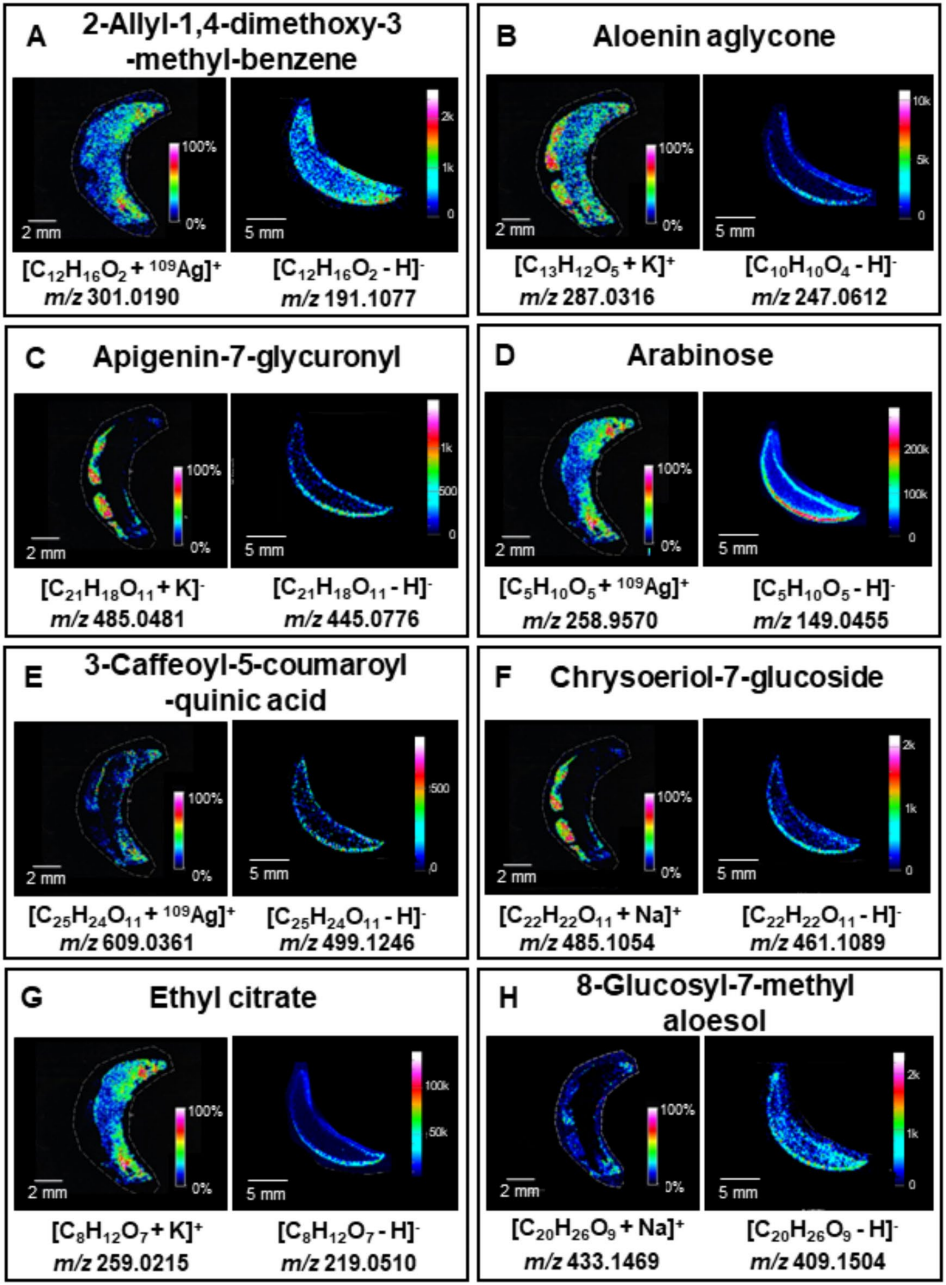
Metabolites identified by both LDI-MSI and LARAPPI/CI-MSI: (**A**) 2-Allyl-1,4-dimethoxy-3-methyl-benzene, (**B**) aloein aglycone, (**C**) apigenin-7-glycuronyl, (**D**) – arabinose, (**E**) 3-caffeoyl-5-coumaroylquinic acid, (**F**) chrysoeriol-7-glucoside, (**G**) ethyl citrate, (**H**) – 8-glucosyl-7-methyl aloesol. For each compound, the left-ion image is for the imprint technique and the right-ion image for ablation.

**Fig. 3:**
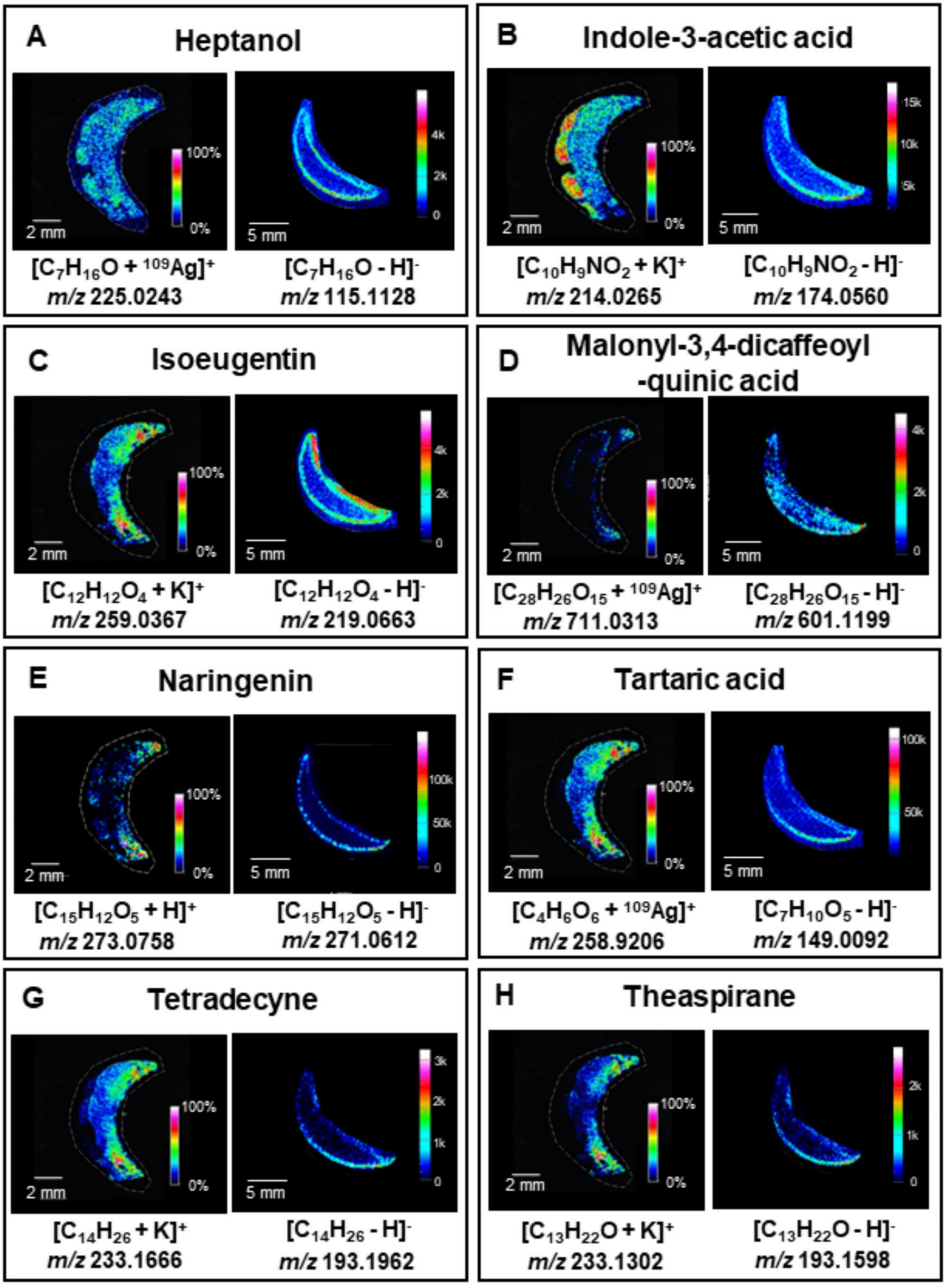
Metabolites identified by both LDI-MSI and LARAPPI/CI-MSI: (**A**) heptanol, (**B**) indole-3-acetic acid, (**C**) – isoeugentin, (**D**) – malonyl-3,4-dicaffeoylquinic acid, (**E**) – naringenin, (**F**) tartaric acid, (**G**) tetradecyne, (**H**) theaspirine. For each compound, the left-ion image is for the imprint technique and the right-ion image for ablation.

Employing two imaging methods allowed for the comparison of MSI results. Twenty-four compounds were found with both MSI methods including eight identified also by the UHPLC-UHRMS method (Figs. 1, 2 and 3), Supplementary Information, Table S2). The comparison of the ion images of the 8 most relevant compounds, which were detected using all three methods and whose presence in *A. vera* has been corroborated by previous scientific publications, is presented in Fig. 1. The remaining common 16 images from both MSI experiments, representing compounds that were putatively identified and are endogenous, are shown in Figs. 2 and 3. Compounds characteristic of *A. vera*, such as aloesin, aloin, and mannose-6-phosphate, were identified using UHPLC-UHRMS (Supplementary Information, Table S1), and their ion images were obtained through ^109^AgNPs-LDI-MSI (Supplementary Information, Fig. S1–5) and LARAPPI/CI-MSI (Supplementary Information, Fig. S6–10) along with MSI of other common metabolites. These images are included in the Supplementary Information, as the study focused on comparing the two imaging methods based on compounds detected by both techniques.

The ion images presented in Fig. 1 demonstrate the spatial distribution and signal intensity of eight metabolites in *A. vera* tissue obtained using two complementary MSI methods: ^109^AgNPs-LDI-MSI and LARAPPI/CI-MSI. The structures of these compounds were confirmed through UHPLC-UHRMS analysis of leaf extracts. The comparison highlights significant differences in the sensitivity and localization capabilities of the two methods.

For 3-coumaric acid (Fig. 1E) and ferulic acid (Fig. 1F), hydroxycinnamic acids that are involved in tissue defense and antioxidative functions as structural components of the cell wall^40^, the spatial distribution differs between methods. While ^109^AgNPs-LDI-MSI shows a strong, localized signal in the adaxial epidermis for both compounds, LARAPPI/CI-MSI demonstrates higher abundance of 3-coumaric acid in the abaxial epidermis and a more even distribution of ferulic acid across the leaf. 3-Coumaric acid plays a key role in the degradation of cinnamate, leading to the formation of citric cycle intermediates such as fumarate and acetyl-CoA^41^, and has been identified in various plant species, including *Agave Angustifolia*^42^, mulberry^43^, olives^44^, and *Ilex kaushue*^45^. The observed differences in spatial distribution between the adaxial and abaxial surfaces may result from differential light exposure, which could influence the accumulation of hydroxycinnamic acids based on their photoprotective roles.

In plants, ferulic acid is synthesized *via* the polyphenolic pathway from caffeic acid or coniferaldehyde and serves as a precursor for lignin biosynthesis, contributing to the formation of guaiacyl and syringyl lignins^46^. Ferulic acid has been previously detected in cereals, such as rice, rye, barley, and maize, particularly in the aleurone cells of seeds^47–49^, and in Aloe tissue extracts analyzed using HPLC^50^ and LC-ESI/MS/MS^51^. Studies using MALDI-MSI^52^ have localized ferulic acid predominantly in the epidermis of plant tissues, including both adaxial and abaxial leaf regions. Similarly, DESI-MSI studies^53^ have shown ferulic acid distribution throughout the leaf in *Salvia miltiorrhiza*. The ion images obtained in this study confirm these findings, showing the highest ferulic acid abundance in the adaxial epidermis for ^109^AgNPs-LDI-MSI and in the abaxial epidermis for LARAPPI/CI-MSI, demonstrating consistency with prior reports.

In this study, gluconic acid (Fig. 1G) exhibits higher signal intensity consistently observed on one side of the leaf cross-section in both MSI methods. ^109^AgNPs-LDI-MSI detected gluconic acid predominantly in the adaxial epidermis with high abundance. In contrast, LARAPPI/CI-MSI revealed a broader distribution concentrated in the same region, but with moderate signal abundance. Both methods showing the highest abundance confined to the epidermal layers. Gluconic acid is a widely occurring compound formed through the oxidation of glucose and serves as a precursor for 6-phosphogluconic acid. Its elevated levels in plants are often associated with microbial activity, including pathogenic infections or symbiotic interactions^54^. Previous studies using MALDI-MSI localized gluconic acid in the outer regions of root tissues in species such as *Panax notoginseng* and *Clitoria fairchildana*^55^, suggesting its role in microbial interactions. In leaves of *Cyclocarya paliurus*, DESI-MSI analysis revealed its accumulation at the junction between the leaf and stem, with lower concentrations in the veins^56^. In *A. vera*, gluconic acid has been previously identified using HPLC-MS/MS^57^. The findings in this study align with prior reports of gluconic acid distribution in plant tissues, although the unique anatomy of *A. vera* leaves, characterized by distinct epidermal regions and a highly hydrated gel layer, urge to consider the specific context for these observations.

Kaempferol (Fig. 1H) was detected with higher intensity on one side of the leaf, specifically in the abaxial epidermis, as revealed by both ^109^AgNPs-LDI-MSI and LARAPPI/CI-MSI. Kaempferol is a flavonoid synthesized in the shikimic acid pathway via intermediate compounds like 4-coumaroyl-CoA, naringenin chalcone, and dihydrokaempferol, with flavanol synthase catalyzing the final step^58^. It is abundant in cruciferous vegetables and tea^59^ and plays a key role in scavenging reactive oxygen species (ROS)^60^, protecting plants from oxidative stress, and regulating ROS and auxin signaling, which influence plant growth. Kaempferol also exhibits antibacterial and antifungal activity^61,62^. In *A. vera*, kaempferol has been previously identified in leaf skin and flower extracts, with higher concentrations in the leaf^63^. Previous MSI studies have localized kaempferol in epidermal tissues in various plants, such as tea and ginkgo leaves^64^, consistent with this study’s findings, which confirm its predominant localization in the abaxial epidermis of *A. vera.*

6-Phosphogluconic acid (Fig. 1I) and ribose 5-phosphate (Fig. 1J) in ^109^AgNPs-LDI-MSI, show the highest intensity in the adaxial epidermis. In contrast, LARAPPI/CI-MSI reveals their highest intensity in the abaxial epidermis, with a broader and more diffuse distribution. 6-Phosphogluconic acid is a substrate in the pentose phosphate pathway (PPP), where it provides phosphate groups for ribulose 5-phosphate, ribose 5-phosphate, and xylulose 5-phosphate through oxidation by 6-phosphogluconate dehydrogenase. Its product, ribose 5-phosphate, is essential for the biosynthesis of key biomolecules, including histidine, tryptophan, NAD^+^, NADP^+^, purines, and pyrimidines^65^. Both compounds are reported here for the first time in *A. vera*, with their presence consistent with their central role in plant metabolism. The pentose phosphate pathway predominantly occurs in plastids, especially chloroplasts, supporting the observed localization of these compounds in the epidermal tissues, where chloroplasts are abundant^66^.

Shikimic acid (Fig. 1K) shows the highest intensity in the abaxial epidermis in both MSI methods. In ^109^AgNPs-LDI-MSI, the signals are most intense in the epidermal region, while in LARAPPI/CI-MSI, the distribution is broader but still strongest in the abaxial region. Shikimic acid is a key intermediate in the shikimate pathway, which links primary and secondary metabolism in plants. It serves as a precursor for aromatic amino acids (phenylalanine, tyrosine, and tryptophan) and other aromatic compounds, including phenylpropanoids and alkaloids. These downstream metabolites play critical roles as structural components of plant cells and as defenses against environmental stressors, such as UV radiation, herbivores, and pathogens^67^. Previous studies using DESI-MSI in *Cyclocarya paliurus* leaves localized shikimic acid primarily in the veins and at the junction between the leaf and stem^56^. Given the identification of numerous secondary metabolites derived from the shikimate pathway in *A. vera* leaves, the occurrence of shikimic acid in this sample is consistent with its known role in plant metabolism.

Sorbifolin (Fig. 1L) in ^109^AgNPs-LDI-MSI is observed in specific regions of the abaxial epidermis. In contrast, LARAPPI/CI-MSI shows a broader distribution with moderate intensity, primarily in the abaxial epidermis as well. Sorbifolin, like most flavonoids, exhibits radical scavenging activity and myeloperoxidase inhibitory effects^68^. It has previously been identified in species such as *Spathelia sorbifolia*^69^, *Thymus herba-barona*^70^, *Mentha x piperita*, and *M. pulegium*^71^, but this study displays its first identification in the Aloe genus. Although sorbifolin has not been extensively studied and no prior MSI analyses have been reported, its consistent localization in the abaxial epidermis suggests a protective role against environmental stressors, consistent with the known functions of flavonoids in plants.

Figures 2 and 3 present 16 compounds detected simultaneously using both MSI techniques. 2-Allyl-1,4-dimethoxy-3-methyl-benzene (Fig. 2A) was distributed throughout the sample in LARAPPI/CI-MSI, with higher intensity in the epidermis, while ^109^AgNPs-LDI-MSI showed it predominantly in the adaxial epidermis, with intensity decreasing centrally. Aloesin aglycone (Fig. 2B) exhibited similar epidermal localization in both methods but was more abundant in the gel region with ^109^AgNPs-LDI-MSI. Apigenin-7-glucuronyl (Fig. 2C) was localized mainly in the abaxial epidermis, with stronger signals in ^109^AgNPs-LDI-MSI.

Arabinose (Fig. 2D) showed broader distribution in LARAPPI/CI-MSI, with the highest signal in the abaxial epidermis, whereas ^109^AgNPs-LDI-MSI localized it at the adaxial epidermis. 3-Caffeoyl-5-coumaroyl-quinic acid (Fig. 2E) was detected in low abundance, particularly on the adaxial side of the leaf, while the signal was absent in some regions of the abaxial epidermis. Chrysoeriol-7-glucoside (Fig. 2F) showed the highest abundance in the abaxial epidermis, with more detailed MSI by ^109^AgNPs-LDI. Ethyl citrate (Fig. 2G) was found on the adaxial side in ^109^AgNPs-LDI-MSI and in the lower abaxial region in LARAPPI/CI-MSI. 8-Glucosyl-7-methyl aloesol (Fig. 2H) was localized to the gel region by LARAPPI/CI-MSI and the epidermis by ^109^AgNPs-LDI-MSI. Heptanol (Fig. 3A) was detected uniformly across the sample by ^109^AgNPs-LDI-MSI, while LARAPPI/CI-MSI localized it at the epidermal region, with no signal in the gel. Indole-3-acetic acid (Fig. 3B) showed higher abundance and signal-to-noise ratio in ^109^AgNPs-LDI-MSI, with uniform distribution in the epidermis and additional detection in the gel contrary to the result of LARAPPI/CI-MSI. Isoeugentin (Fig. 3C) was concentrated in the adaxial epidermis in both methods, with lower abundance detected across other epidermal regions by LARAPPI/CI-MSI. Malonyl-3,4-dicaffeoyl-quinic acid (Fig. 3D) was detected at low intensity in the adaxial and abaxial epidermis by ^109^AgNPs-LDI-MSI but was exclusively localized in the gel and inner epidermis by LARAPPI/CI-MSI. The final compounds—naringenin (Fig. 3E), tartaric acid (Fig. 3F), tetradecyne (Fig. 3G), and theaspirane (Fig. 3H) exhibited complementary patterns. ^109^AgNPs-LDI-MSI showed the highest intensity in the adaxial epidermis, while LARAPPI/CI-MSI localized them to the abaxial epidermis, with comparable overall intensities. Naringenin showed stronger signals and a better signal-to-noise ratio in ^109^AgNPs-LDI-MSI, with some detection in the lower leaf region.

The detected compounds presented in Figs. 2 and 3 play key roles in the metabolic and defense mechanisms of *A. vera*. Flavonoids such as apigenin-7-glucuronyl and chrysoeriol-7-glucoside provide antioxidant protection, while aloesin aglycone and 8-glucosyl-7-methyl aloesol are involved in gel maintenance and hydration regulation. Indole-3-acetic acid, a plant hormone, regulates growth and development, and organic acids like tartaric acid support pH balance and metabolic pathways. Compounds such as naringenin and 3-caffeoyl-5-coumaroyl-quinic acid are intermediates in phenolic biosynthesis, enhancing stress resistance and structural integrity. These metabolites reflect *A. vera’s* adaptations to environmental stress and its unique biological functions.

### Analysis of *A. vera* extracts using the UHPLC-UHRMS method

UHPLC-UHRMS analysis provided 304 identified compounds, 137 in the negative mode and 226 in the positive mode. The results involved essential compounds such as amino acids, organic acids, fatty acyls and other lipids, but a substantial amount of compounds identified were secondary plant metabolites. Among them the main groups would be cinnamic acid derivatives, such as coumaric acid and ferulic acid, polyphenolic secondary metabolites, flavones, flavanones, flavonoles and their glycosides, and metabolites of the shikimate pathway. Additionally, many compound widely associated with the Aloe genus were also detected. Those compounds are aloeresin A, aloeresin D, aloesin and aloin. Except for aloeresin D, all of those compounds were detected in negative mode. Both aloeresin A and aloeresin D were detected in the positive mode. Aloeresin A and aloeresin D have been identified in 7 and 14 aloe species respectively, incudin *A. vera*^72,73^. Both aloesin and aloin have been identified in more than 20 aloe species. Except for aloin, those compounds could be classified as chromones, common plant metabolites. The UHPLC-UHRMS analysis results are summarized in Supplementary Information, Table S1 and Fig. S11.

### Comparison of ^109^AgNPs-LDI-MSI and LARAPPI/CI-MSI

In both methods we employed, preliminary sample preparation was minimal and did not require embedding the tissue in a cryosectioning medium or coating with an organic matrix, as is necessary in MALDI-MSI^74^. In the imprinting method we used, the sample was pressed against the steel target surface for a few seconds, during which chemical compounds were transferred from the sample to the target plate’s surface. Its main advantages besides simplicity and rapidness is reduced risk of sample damage, and minimal amount of artifacts. Unlike traditional methods, it mitigates the risks associated with direct sample exposure to high vacuum conditions during analysis, such as dehydration, mechanical stress, or redistribution of analytes. The method is highly effective for low molecular weight compounds (< 1500 Da), providing low background noise, high sensitivity (up to attomole level), and precise mass accuracy through multi-point calibration with silver-109 ions. However, the imprint method has limitations, particularly with very hard or soft tissues, which can affect imprint quality and metabolite detection^25^.

When preparing a sample for MSI using the LARAPPI/CI method, the process is significantly simplified, as it only requires placing tissue of nearly any thickness onto the target plate. The sample is frozen using a Peltier module, ensuring stability and immobility throughout the analysis.

Tissue thickness plays a critical role in MSI sample preparation, as it directly influences signal intensity. A study using MALDI-ToF MS on FFPE tissue microarrays demonstrated higher signal intensities for 1 μm sections compared to 5 μm sections, highlighting the sensitivity advantages of thinner samples^75^. However, achieving such thin sections consistently poses challenges due to technical issues like floating and rolling. In traditional MSI techniques, such as MALDI, thin tissue Sects. (5–20 μm) enhance spatial resolution by reducing signal overlap between layers and enabling efficient ionization. In contrast, thicker sections can lead to signal interference from deeper layers, incomplete analyte desorption, and uneven matrix application, which results in localized signal variations and reduced data quality. Furthermore, analyte migration during preparation, caused by mechanical stress during sectioning or water evaporation during drying, can distort the native compound distribution^76^.

The LARAPPI/CI system employs a distance sensor functioning as a surface profilometer, enabling precise control of the ablation height during experiments, which ensures accurate removal of tissue layers. The use of a flat-top laser beam profile, achieved through diffractive optical elements, ensures uniform material removal and reduces the formation of artifacts. This beam shape produces shallow, rectangular ablation craters. Unlike conical beams that create deeper craters and can result in overlapping ablation zones, the flat-top profile ensures precise material removal with minimal distortion.

In the imprint method, sample thickness does not significantly affect the resolution of MSI, as the key process is the transfer of chemical compounds from the sample surface to the plate, rather than the analysis of the entire sample volume. It is possible to achieve instruments maximum resolution, however it is not practical for larger objects as imaging time is very long. The common practice in our surface transfers is to observe imprint in high magnification optical microscope to assess practical resolution of the transfer and set MSI resolution accordingly.

The ^109^AgNPs-LDI-MSI method employs metal nanoparticles as a surface-assistance, utilizing their localized surface plasmon resonance (LSPR) to enhance absorptive properties. This phenomenon increases the signal intensity for molecules absorbed on the nanoparticle surface, making it particularly effective for analyzing small molecules. In the context of *A. vera* leaves, this method demonstrates a higher sensitivity, enabling the detection of compounds not visible with LARAPPI/CI-MSI. For example, ^109^AgNPs-LDI-MSI reveals the presence of some compounds in lower concentrations in the inner part of the leaf, a feature that complements the more surface-oriented imaging of LARAPPI/CI-MSI.

The comparison of the two MSI methods highlights that most detected compounds are concentrated in the epidermis, as expected given the high water content (95–98%) of the *A. vera* gel. The post-analysis image of the leaf after LARAPPI/CI-MSI analysis suggests that the ablation was uniformly conducted across the whole leaf slice. Both methods operate in a similar *m/z* range and detect a comparable number of amino acids and carbohydrates. However, LDI-MSI excels in the identification of lipid compounds, detecting over twice as many as LARAPPI/CI-MSI, whereas LARAPPI/CI-MSI outperforms in detecting aromatic compounds, phenolics, and organic acids. Importantly, LARAPPI/CI-MSI provides much higher spectral resolution, allowing for much higher quality of identification. By contrast, LDI-MSI provides less extensive data for pathway mapping but excels in detecting lipids and other small molecules absorbed on nanoparticle surfaces. Despite these differences, the spatial localization of compounds between the two methods is largely consistent, underscoring their reliability. The complementary strengths of these techniques enable a more comprehensive analysis of plant tissues, with LARAPPI/CI-MSI offering superior spatial resolution and enhanced precision for plant-specific metabolites and pathway mapping, while LDI-MSI provides deeper insight into lipid distributions and small molecule detection. Together, these methods contribute valuable information on the metabolic activity and structural organization of *A. vera* leaves, with minimal compromise in spatial resolution or accuracy even in highly hydrated tissues.

### Metabolic pathways based on the identified compounds

MSI is a key tool for analyzing metabolic pathways in plant tissues, providing high-resolution spatial mapping of metabolite distribution. Ion images generated by MSI reveal the localization of metabolites, which can be used to infer metabolic activity and reconstruct pathways based on spatial correlations between metabolites and their products. MSI offers semi-quantitative analysis by comparing relative signal intensities, which indicate the relative abundance of metabolites across tissue regions. While not fully quantitative, these data provide valuable insights into the localization and dynamics of metabolic processes^77^. Integrating MSI with other “omics” approaches enables a comprehensive understanding of plant metabolism, revealing spatial relationships between metabolites and their roles in stress responses and metabolic interactions^78,79^.

For pathway analysis, LARAPPI/CI-MSI was selected over ^109^AgNPs-LDI-MSI due to its much higher spectral resolution and superior ability to detect plant-specific metabolites, including aromatic compounds, phenolics, and organic acids. The enhanced resolution of QToF instrument used for LARAPPI/CI-MSI enables more detailed visualization of compound localization and intensity, which is essential for accurately reconstructing metabolic pathways and identifying the spatial organization of key reactions.

All identified compounds were used to determine metabolic pathways occurring in *A. vera* leaves using MetaboAnalyst version 6.0. The best fit based on the match status and continuity of the pathway based on the ion images was obtained for pathways of flavonoid biosynthesis, pentose phosphate pathway, and phenylpropanoid biosynthesis. The full results of the matching process are presented in Supplementary Information Table S3.

**Fig. 4:**
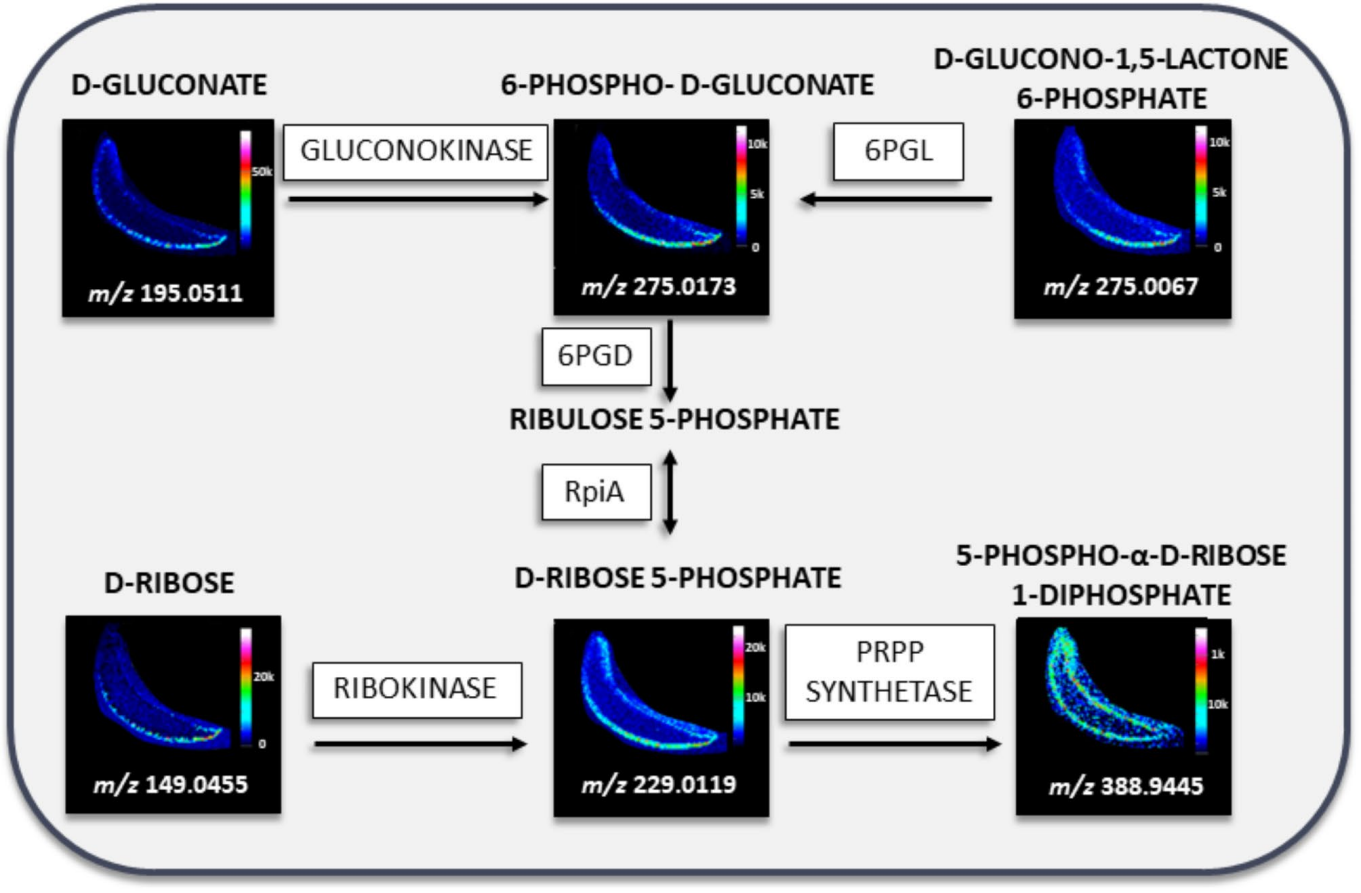
Ion images obtained by LARAPPI/CI-MSI of compounds present in PPP. The fragment of the pathway was presented for comparison of the location of compounds in the pathway in *A. vera* leaf; 6PGL – 6-phosphogluconolactonase, 6PGD – 6-phosphogluconate dehydrogenase, rpiA – ribose-5-phosphate isomerase A.

The compounds identified as part of the pentose phosphate pathway (PPP) using LARAPPI/CI-MSI (Fig. 4) include D-gluconate, 6-phospho-D-gluconate, D-glucono-1,5-lactone 6-phosphate, D-ribose, D-ribose 5-phosphate, and 5-phospho-α-D-ribose 1-diphosphate. These compounds are distributed primarily in the abaxial epidermis, with D-gluconate exhibiting the highest signal intensity. 6-Phospho-D-gluconate and D-glucono-1,5-lactone 6-phosphate show small regions of higher intensity in the adaxial epidermis, suggesting a potential metabolic connection between them. In the pathway, 6-phospho-D-gluconate is metabolized by 6PGD to produce ribulose 5-phosphate, which is further transformed into D-ribose 5-phosphate by RpiA. Both D-ribose 5-phosphate and its precursor, D-ribose, were detected, with D-ribose 5-phosphate showing higher abundance and a more extensive distribution in the epidermis, likely due to multiple synthesis sources. Its product, 5-phospho-α-D-ribose 1-diphosphate, was identified with much lower abundance and a spatial distribution similar, but not identical, to its precursor. PPP is a critical pathway in plants, producing metabolites required for nucleotide biosynthesis and contributing to antioxidant activity through NADPH production^80^. NADPH plays a key role in redox reactions involved in glutathione and thioredoxin activity^81^. In plants, PPP is more active in leaf tissues than in roots, potentially correlating with photosynthetic activity. However, studies suggest that stromal oxidative PPP is inactive during photosynthesis, implying that PPP activity is regulated by non-photosynthetic processes^82^. The higher concentration of compounds such as D-gluconate, 6-phospho-D-gluconate, and D-ribose in the abaxial epidermis and regions with less light exposure suggests that light availability may influence their abundance^66^.

**Fig. 5:**
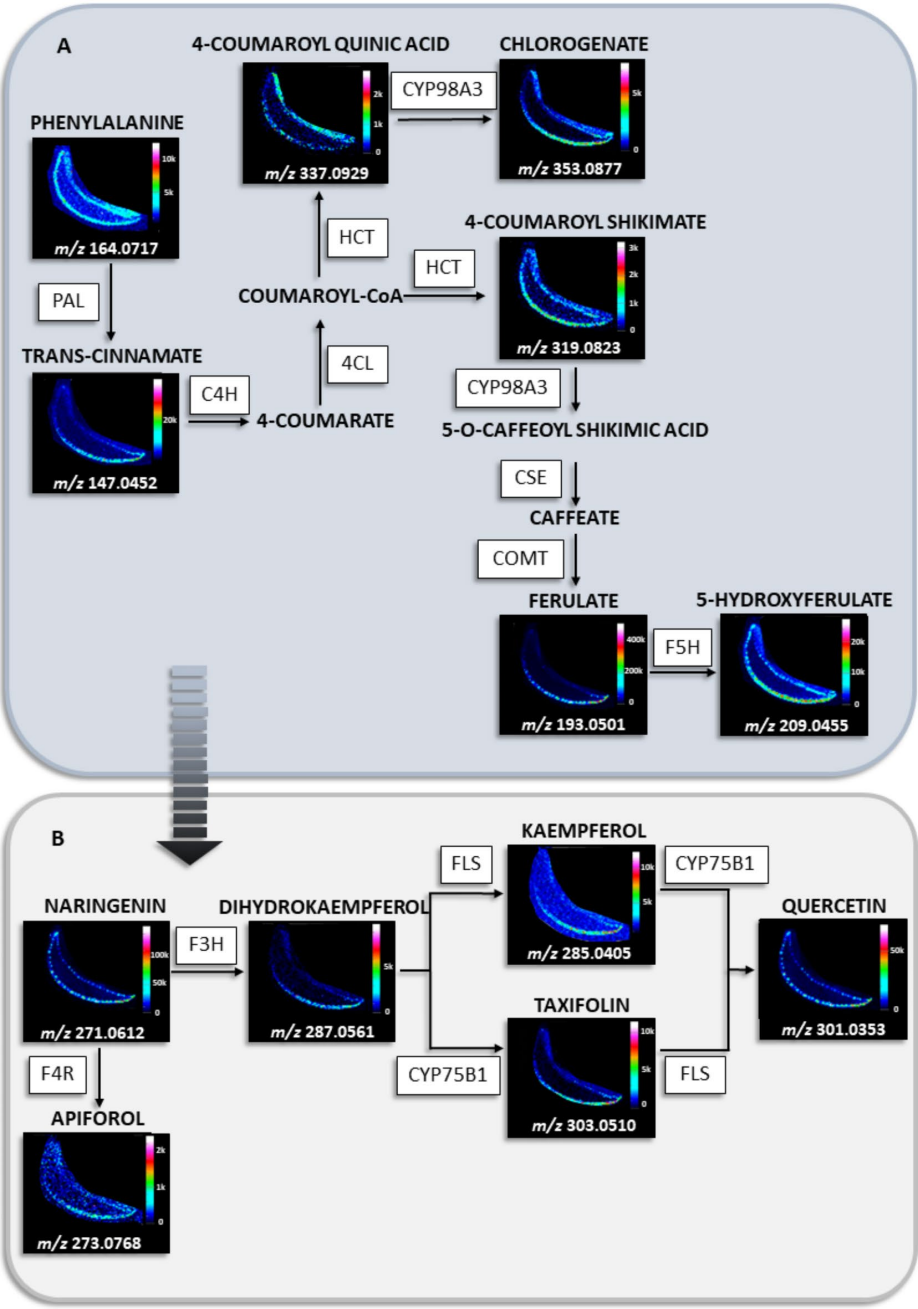
Ion images obtained by LARAPPI/CI-MSI of compounds present in A - phenylpropanoid biosynthesis pathway and B - flavonoid biosynthesis pathway. The fragments of the pathways were presented for comparison of the location of compounds in the pathway in *A. vera* leaf; 4CL − 4-coumarate–CoA ligase, C4H – trans-cinnamate 4-monooxygenase, COMT – caffeic acid 3-O-methyltransferase, CSE - caffeoylshikimate esterase, CYP75B1 - flavonoid 3’-monooxygenase, CYP98A (C3’H) − 5-O-(4-coumaroyl)-D-quinate 3’-monooxygenase, F3H – flavanone 3-hydroxylase, F4R – flavonone 4-reductase, F5H – ferulate-5-hydroxylase, FLS – flavonol synthase, HCT – shikimate O-hydroxycinnamoyltransferase, PAL – phenylalanine ammonia-lyase.

The ion images obtained using LARAPPI/CI-MSI revealed the spatial distribution of key metabolites in the phenylpropanoid biosynthesis pathway (Fig. 5A), including phenylalanine, trans-cinnamate, 4-coumaroyl quinic acid, chlorogenate, 4-coumaroyl shikimate, ferulate, and 5-hydroxyferulate. Phenylalanine, abundantly present throughout the epidermal region, serves as the starting compound in this pathway. Through enzymatic deamination by PAL, it is converted to trans-cinnamate, which is further hydroxylated by C4H to form 4-coumarate. This compound undergoes activation by 4CL, producing coumaroyl-CoA, which acts as a precursor for downstream metabolites, including 4-coumaroyl quinic acid and 4-coumaroyl shikimate.

Chlorogenate, derived from 4-coumaroyl quinic acid via CYP98A3, is a key antioxidant and defense compound, abundant in the lower abaxial epidermis, similar to its precursor. Ferulate, another significant product in this pathway, exhibited the highest abundance among the identified compounds, concentrated in the lower leaf regions. This suggests its crucial role in oxidative stress protection. The final metabolite, 5-hydroxyferulate, was more evenly distributed across the epidermis but with lower abundance than its precursor. The phenylpropanoid pathway is pivotal for plant defense, contributing to the synthesis of lignins and flavonoids^83^. Lignins reinforce plant structure, while flavonoids provide UV protection and regulate growth^84,85^. The impact of UV-B radiation has been linked to changes in phenylpropanoid-derived compounds, with extended exposure reducing ferulic acid and hydroxycinnamic acid concentrations^86,87^, both of which influence photosynthetic activity^88,89^. In the flavonoid biosynthesis pathway (Fig. 5B), key metabolites, including naringenin, apiforol, dihydrokaempferol, kaempferol, taxifolin, and quercetin, were identified. Naringenin, present in high abundance in the inner epidermis, serves as the central branching point, leading to the synthesis of protective flavonoids. It is converted to dihydrokaempferol by F3H, which then forms kaempferol via FLS or taxifolin via CYP75B1 ^93^. Kaempferol and taxifolin are precursors to quercetin, a major flavonoid distributed similarly to naringenin, highlighting its importance in this pathway. The increased abundance of flavonoids in specific epidermal regions correlates with their role in plant defense and UV protection^91,92^. While physical handling can impact compound detection, the observed distribution patterns are consistent with active metabolic pathways rather than sample preparation artifacts^93^. Together, the phenylpropanoid and flavonoid biosynthesis pathways illustrate the plant’s adaptive mechanisms to environmental stress and its metabolic coordination across different tissue regions.

## Conclusions

The study allowed for the identification of key *A. vera* metabolites, such as phenylpropanoids and flavonoids. The identified compounds show properties that confirm the usefulness of Aloe extracts in medicinal, food, and cosmetic uses. The ion images obtained from the two laser ablation-based MSI methods proved that the majority of the compounds were identified in the skin or latex regions of the plant leaf. Further analysis of potential metabolic pathways based on acquired ion images proved the metabolic processes identified undergo in the epidermal part of the *A. vera* leaf. The specific structure of the leaf seems to confirm acquired results. The difference in the used desorption/ionization techniques, as well as sample vs. imprint analysis, showed slight discrepancies in the location of the compounds identified with both methods. Since the majority of the compounds identified with ion images showing their occurrence in the gel region have not been previously identified in that part of the leaf the probable explanation is the effect of drifting of compounds during imprinting due to high moisture levels. The novel LARAPPI/CI-MSI technique proved to be preferential in the detection of plant-related metabolites, and the ^109^AgNPs-LDI-MSI performed better in lipid detection. The integration of three distinct analytical platforms, ^109^AgNPs-LDI-MSI, LARAPPI/CI-MSI, and UHPLC-UHRMS, enabled a comprehensive and highly efficient visualization of metabolites in an aloe leaf cross-section, providing deeper insights into its metabolic processes. This multi-platform approach facilitated a more detailed understanding of the tissue’s biochemical landscape.

## Electronic supplementary material

Below is the link to the electronic supplementary material.

Supplementary Material 1

## Data Availability

The data sets generated during and/or analyzed during the current study are available from the corresponding author upon request and in the RepOD open data repository (DOI: 10.18150/TNVBKJ).
