# Supplementary material for: Analysis of the spatial distribution of metabolites in Aloe vera leaves by mass spectrometry imaging and UHPLC-UHRMS
Source: Sci Rep. 2025 Jan 28;15:3502. doi: 10.1038/s41598-025-88144-8 (PMC11775111; doi:10.1038/s41598-025-88144-8)
Supplement: Supplementary file 1 — Supplementary Material 1 [file 41598_2025_88144_MOESM1_ESM.docx]

Supplementary Information

**Analysis of the spatial distribution of metabolites in *Aloe vera* leaves by mass spectrometry imaging and UHPLC-UHRMS**

Sumi Krupa^1^, Tomasz Ruman^2^, Wiktoria Szuberla^2^, Joanna Nizioł*^3^

^1^Doctoral School, Rzeszów University of Technology, 8 Powstańców Warszawy Ave., 35-959 Rzeszów, Poland.

^2^Department of Inorganic and Analytical Chemistry, Faculty of Chemistry, Rzeszów University of Technology, 6 Powstańców Warszawy Ave., Rzeszów, 35-959, Poland

^3^Department of Polymers and Biopolymers, Faculty of Chemistry, Rzeszów University of Technology, 6 Powstańców Warszawy Ave., Rzeszów, 35-959, Poland

* Corresponding author:

**Table of Contents:**

**Fig. S1. – Fig. S5.**  ^109^AgNPs-LDI-MSI ion images with identified compound ions.

**Fig. S6. – Fig. S10.** LARAPPI/CI-MSI ion images with identified compound ions.

**Table S1.** Results of UHPLC-HRMS analysis of *Aloe vera* extracts.

**Fig. S11** The UHPLC-HRMS chromatograms for selected compounds

**Table S2.** Metabolites localized in Aloe vera samples by both MSI methods used in the study.

**Table S3.** Pathway analysis results.


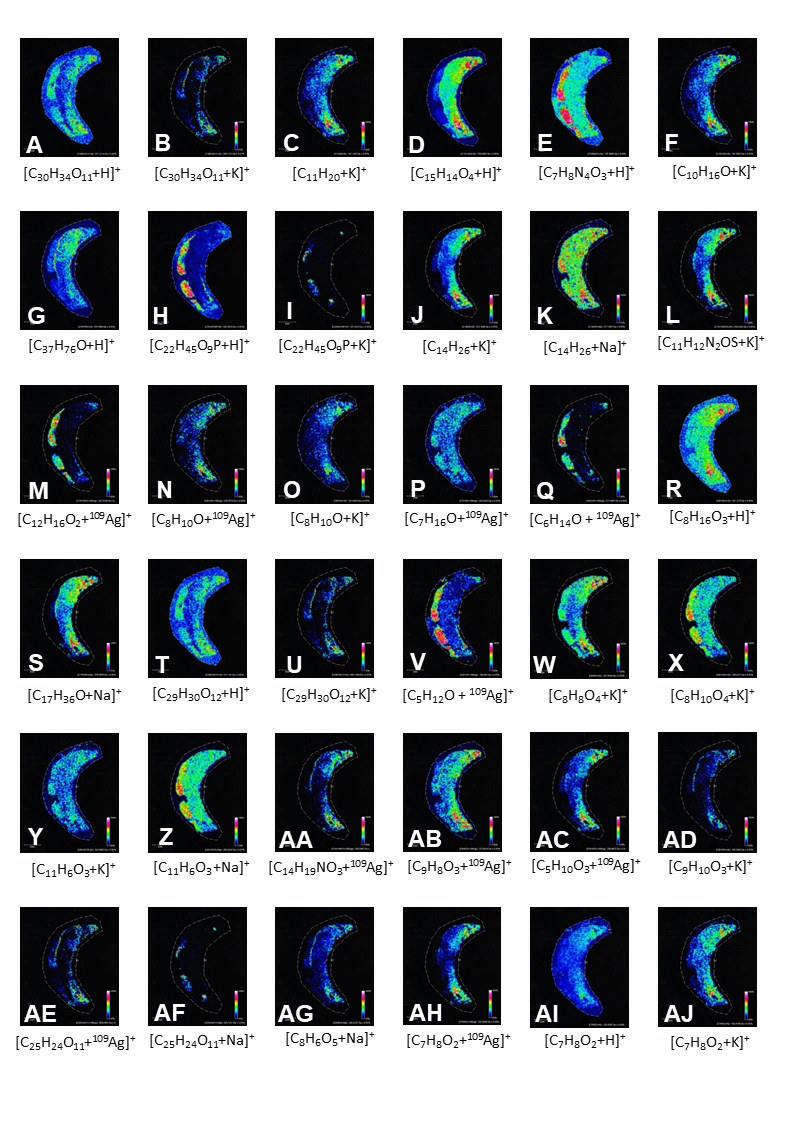


**Fig. S1.** ^109^AgNPs-LDI-MSI ion images of compounds identified by UHPLC-UHRMS. **A-D** - unidentified, **E** - 1,3-Dimethyluric acid, **F, G** - unidentified, **H, I** - 1-Palmitoyl-2-hydroxy-sn-glycero-3-phospho-(1'-rac-glycerol), **J-W** - unidentified, **X** - 3,4-Dihydroxyphenylglycol, **Y, Z, AA** – unidentified, **AB** - Coumaric acid, **AC** - 3-Hydroxyisovaleric acid, **AD** - 3-Phenyllactic acid, **AE, AF** - unidentified, **AG** - 4-Hydroxyisophthalic acid, **AH, AI, AJ** - 4-Methylcatechol.


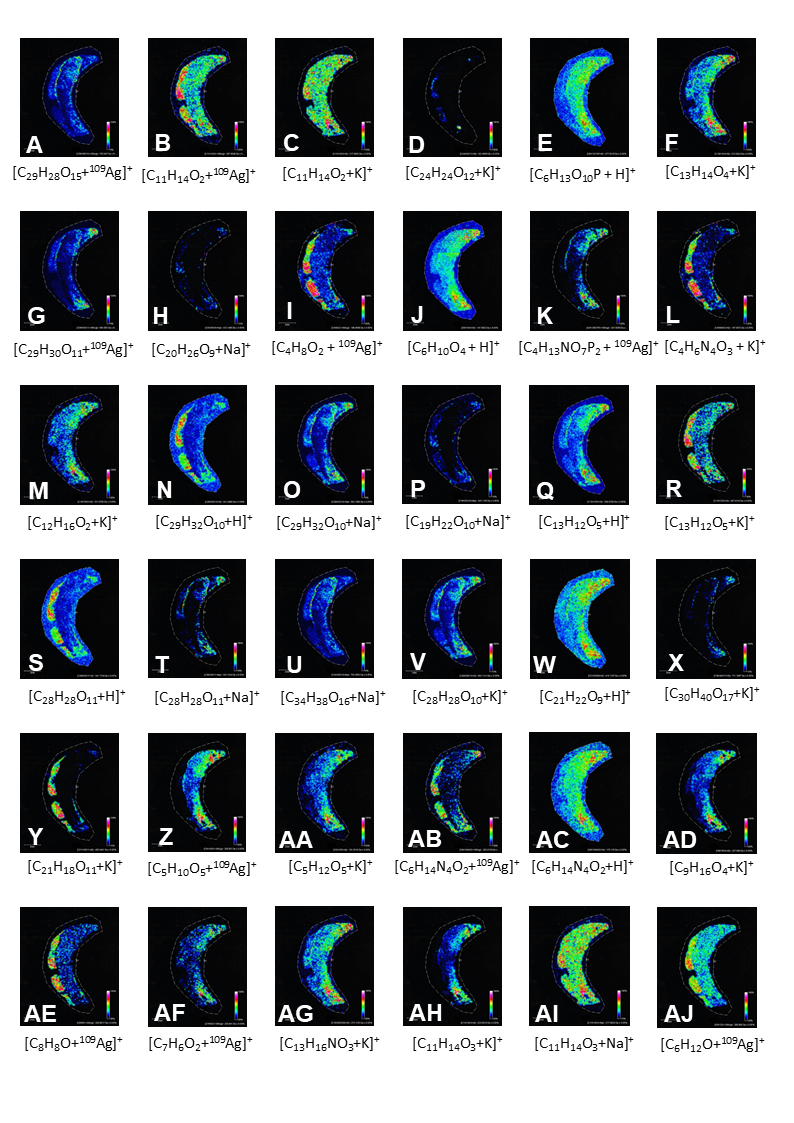


**Fig. S2.** ^109^AgNPs-LDI-MSI ion images of compounds identified by UHPLC-UHRMS. **A** - unidentified, **B, C** - 5-Phenylvaleric acid, **D** - unidentified, **E** - 6-Phosphogluconic acid, **F** - 7-Hydroxy-3-(2-hydroxypropyl)-5-methylisochromen-1-one, **G**, **H** - unidentified, **I** – Acetoin, **J** - Adipic acid, **K** - Alendronate sodium trihydrate, **L** – Allantoin, **M**-**R** - unidentified, **S, T** - Aloeresin A, **U-AA** - unidentified, **AB, AC** – Arginine, **AD** – Azelaic acid, **AE** – unidentified, **AF** – Benzoic acid, **AG-AJ** – unidentified.


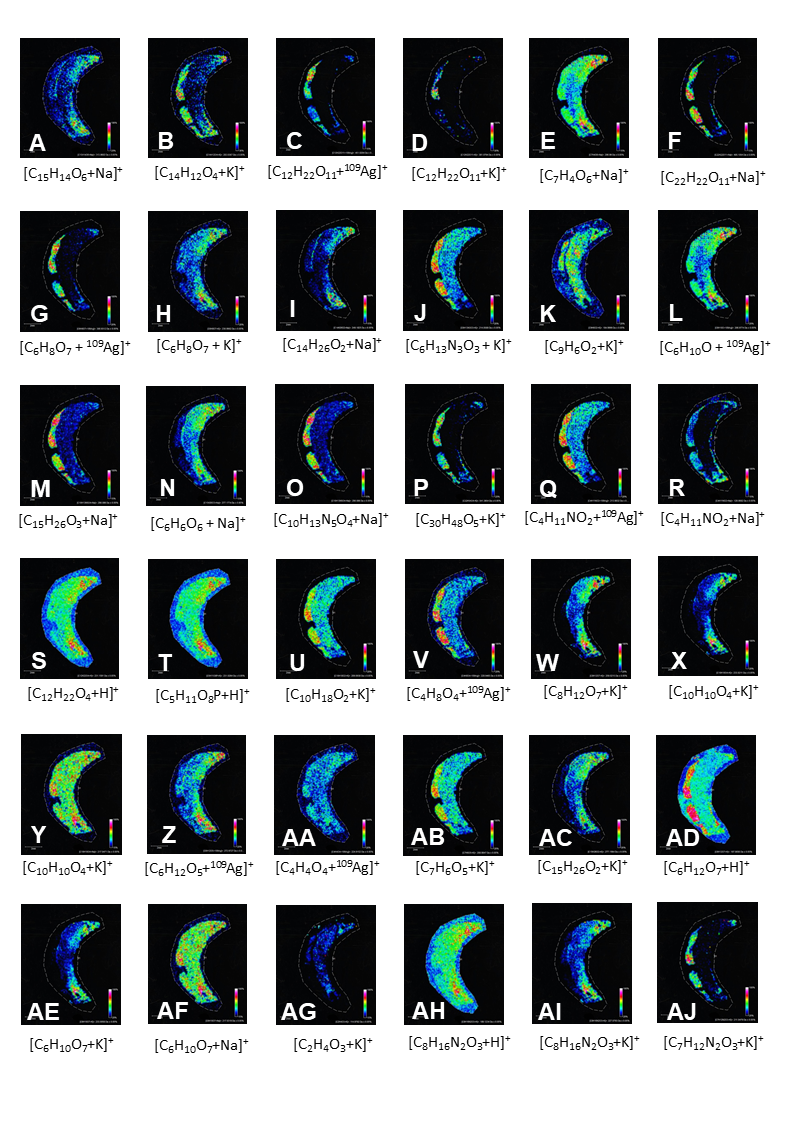


**Fig. S3.** ^109^AgNPs-LDI-MSI ion images of compounds identified by UHPLC-UHRMS. **A**, **B** – unidentified, **C, D** – Cellobiose, **E** – Chelidonic acid, **F** - unidentified, **G, H** – Citric acid, **I** – unidentified, **J** – Citrulline, **K** – Coumarin, **L** – Cyclohexanone, **M** – Dehydroascorbic acid, **N**– unidentified, **O** – Deoxyguanosine, **P** – unidentified, **Q, R** – Diethanolamine, **S** - Dodecanedioic acid, **T** - D-Ribose 5-phosphate, **U** - unidentified, **V** – Erythrose, **W** - unidentified, **X, Y** – Ferulic acid, **Z**-**AC** – unidentified, **AD** – Gluconic acid, **AE, AF**, **AG** – unidentified, **AH, AI** – Glycyl-L-leucine, **AJ** – Glycylproline.


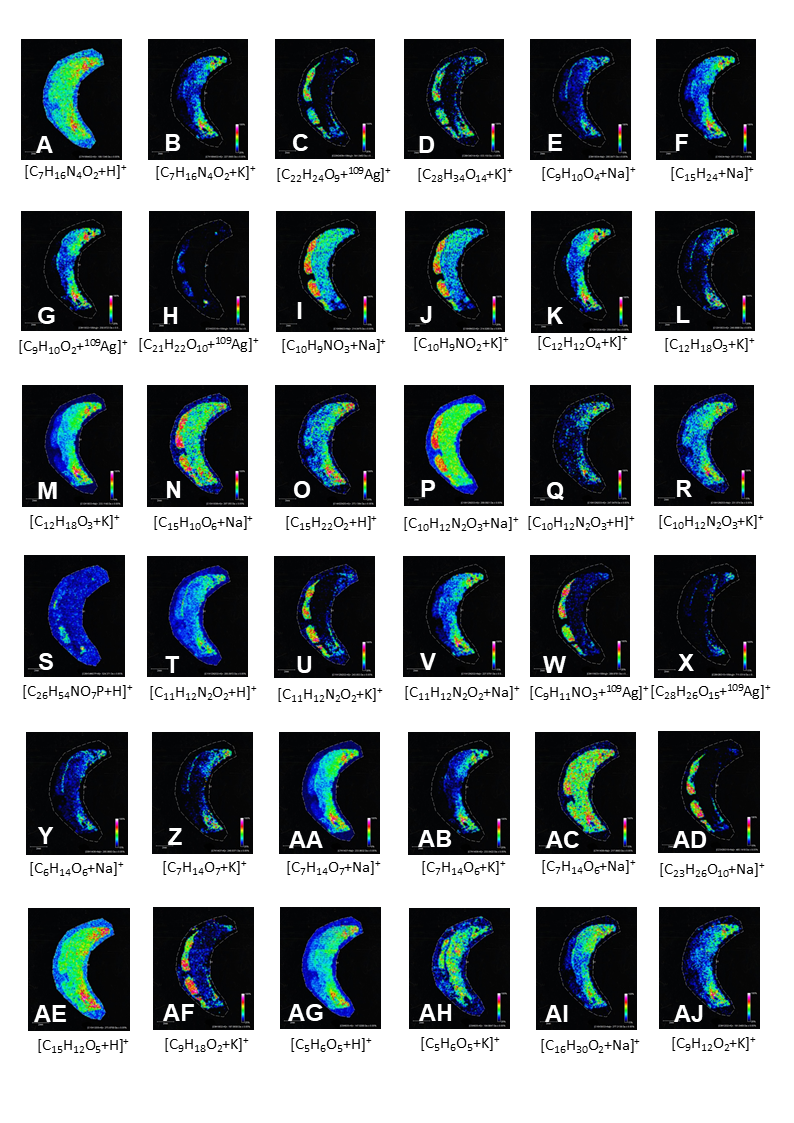


**Fig. S4.** ^109^AgNPs-LDI-MSI ion images of compounds identified by UHPLC-UHRMS **A,** **B** – Homo-L-arginine, **C**-**F** – unidentified, **G** – Hydrocinnamic acid, **H** – unidentified, **I** – Hydroxyindoleacetic acid, **J**, **K** – unidentified, **L, M** – Jasmonic acid**, N** – Kaempferol, **O** – unidentified, **P, Q, R** - L-Kynurenine, **S** – unidentified, **T, U, V** - L-Tryptophan, **W** – L-Tyrosine, **X** - unidentified, **Y** – Mannitol, **Z-AC** - unidentified, **AD** - Methylnissolin-3-O-glucoside, **AE**, **AF** – unidentified, **AG, AH** – Oxoglutaric acid, **AI**, **AJ** - unidentified.


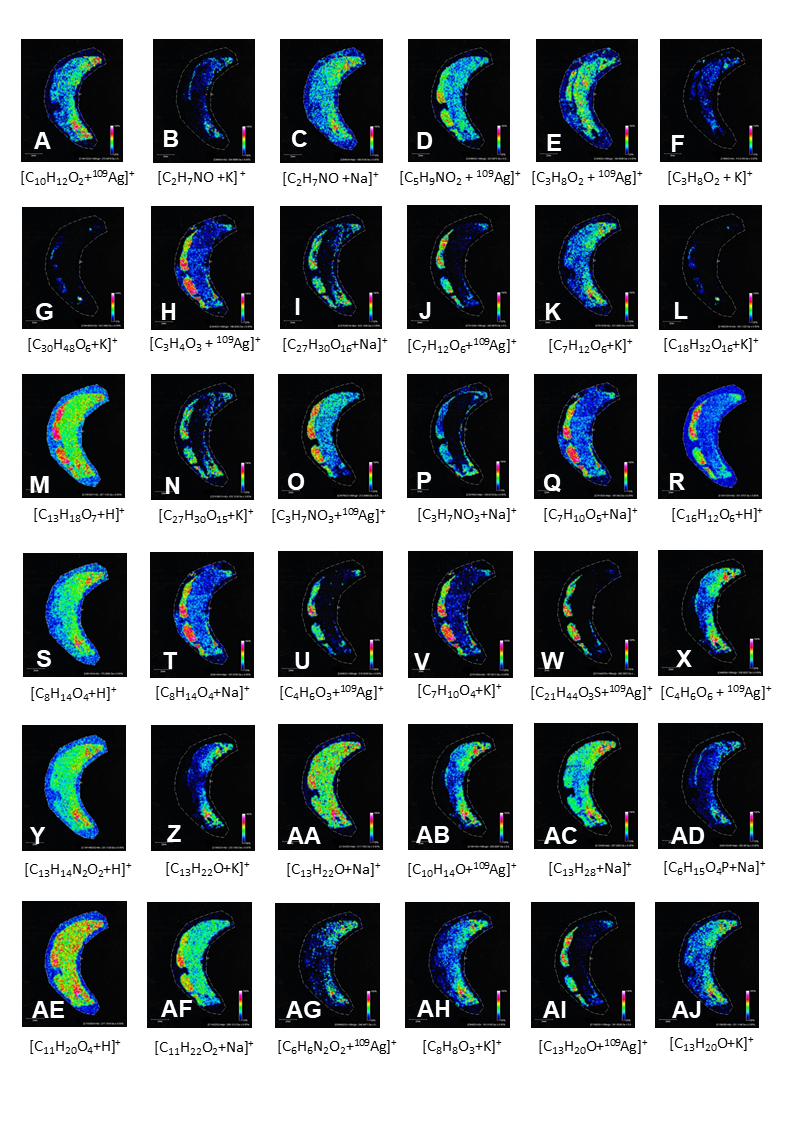


**Fig. S5.** ^109^AgNPs-LDI-MSI ion images of compounds identified by UHPLC-UHRMS **A**, **B,** **C** – unidentified, **D** – Proline, **E, F** – unidentified,  **G** - Pygenic acid C, **H**, **I** – unidentified, **J, K** – Quinic acid, **L** – Raffinose, **M** – Salicin, **N** – Kaempferol-O-rutinoside, **O, P** – Serine, **Q** – Shikimic acid, **R** – Sorbifolin, **S, T** – unidentified, **U** – Succinic acid semialdehyde, **V** – Succinylacetone, **W**, **X** – unidentified, **Y** – Tetrahydroharman-3-carboxylic acid, **Z-AC** – unidentified, **AD** – Triethylphosphate, **AE, AF** – Undecanedioic acid, **AG**– Urocanic acid, **AH**, **AI, AJ** – unidentified.


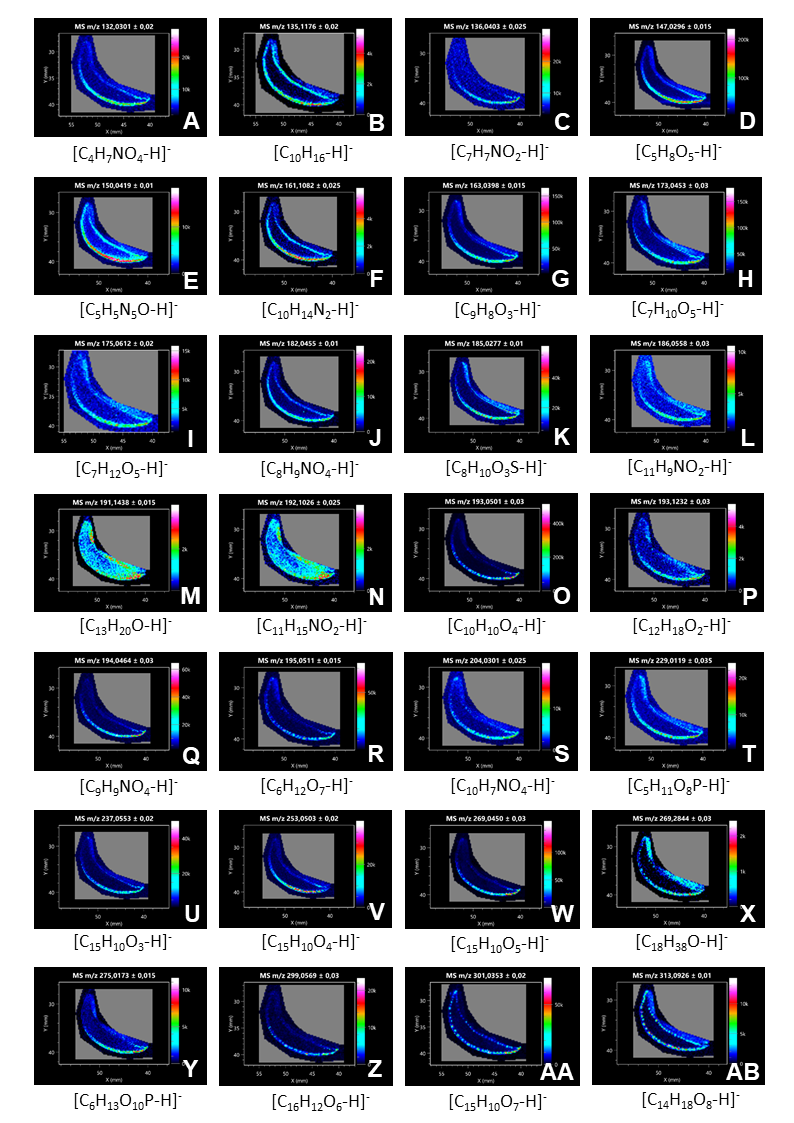


**Fig. S6.** LARAPPI/CI-MSI ion images of compounds identified by UHPLC-UHRMS. **A** – Aspartic acid, **B** – Limonene, **C** -Trigonelline, **D** – 2-Hydroxy-2-methylbutanedioic acid, **E** – Isoguanine, **F** – unidentified, **G** – 3-Coumaric acid, **H** – Shikimic acid, **I** – 2-Isopropylmalic acid, **J** – 4 – Pyridoxic acid, **K** – 3,4-Dimethylbenzene-1-sulfonic acid, **L** – 3-Indoleacrylic acid, **M**, **N** – unidentified, **O** – Trans-Ferulic acid, **P** - unidentified, **Q** - 3-Hydroxyhippuric acid, **R** - Gluconic acid, **S** - Xanthurenic acid, **T** - D-Ribose 5-phosphate, **U** - 4'-Hydroxyflavone, **V** - 3',4'-Dihydroxyflavone, **W** - unidentified, **X** – Octadecanol, **Y** - 6-Phosphogluconic acid, **Z** – Sorbifolin, **AA** – Quercetin, **AB** - (4-(β-D-Glucopyranosyloxy)phenyl)acetic acid.


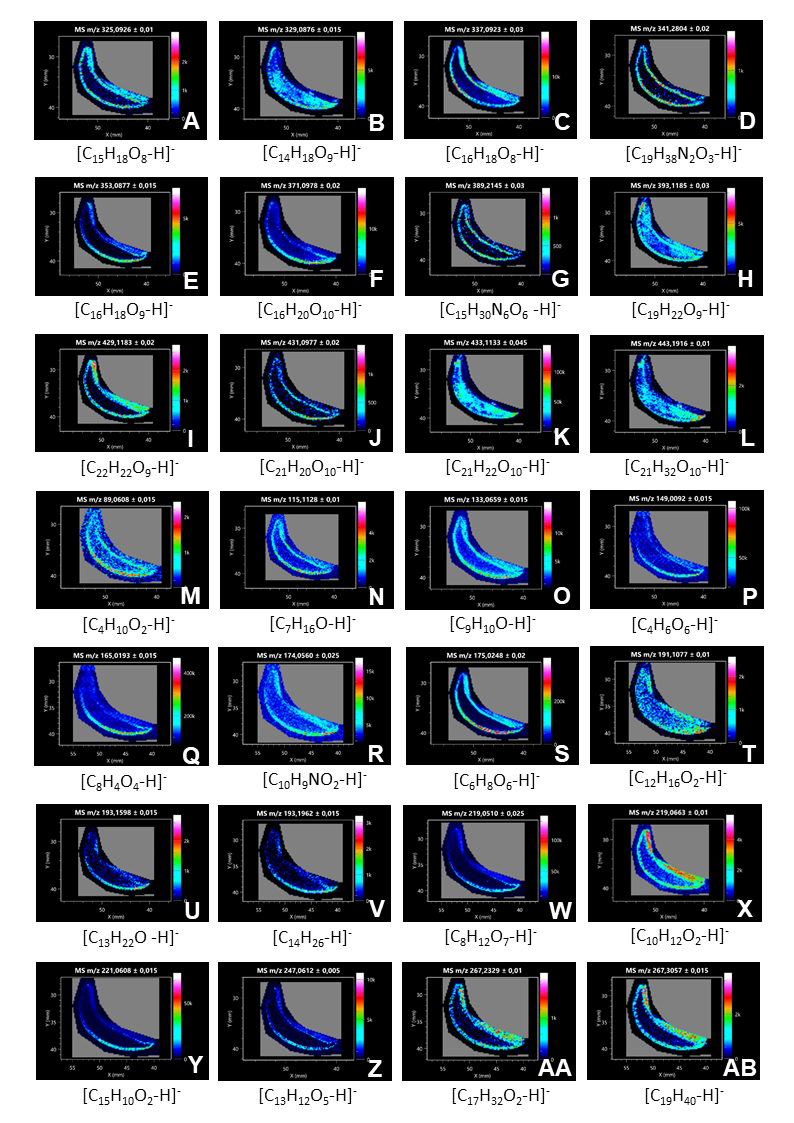


**Fig. S7.** LARAPPI/CI-MSI ion images of compounds identified by UHPLC-UHRMS. **A** - Cis-Melilotoside, **B** - 4-(Hexopyranosyloxy)-3-methoxybenzoic acid, **C** - 3-O-p-Coumaroylquinic acid, **D** - Cocamidopropyl Betaine, **E** - Chlorogenic acid, **F** - 3-(Benzoyloxy)-2-hydroxypropyl-β-D-glucopyranosiduronic acid, **G** - unidentified, **H** – Aloesin, **I** - Formononetin-7-O-glucoside, **J** - 5,7-Dihydroxy-2-(4-hydroxyphenyl)-6-[3,4,5-trihydroxy-6-(hydroxymethyl)oxan-2-yl]chromen-4-one, **K** - 1,8,10-Trihydroxy-3-(hydroxymethyl)-10-[3,4,5-trihydroxy-6-(hydroxymethyl)oxan-2-yl]anthracen-9-one, **L** - 1-O-((2E,4E)-5-((1R,3S,5S,8S)-3,8-Dihydroxy-1,5-dimethyl-6-oxabicyclo[3.2.1]octan-8-yl)-3-methylpenta-2,4-dienoyl)-β-D-glucopyranose, **M**-**Q** - unidentified, **R** - Indole-3-acetic acid, **S**, **T** - unidentified, **U** - Ferulic acid, **V** – unidentified, **W**-**Z** - unidentified, **AA**- Heptadecenoic acid, **AB** – unidentified.


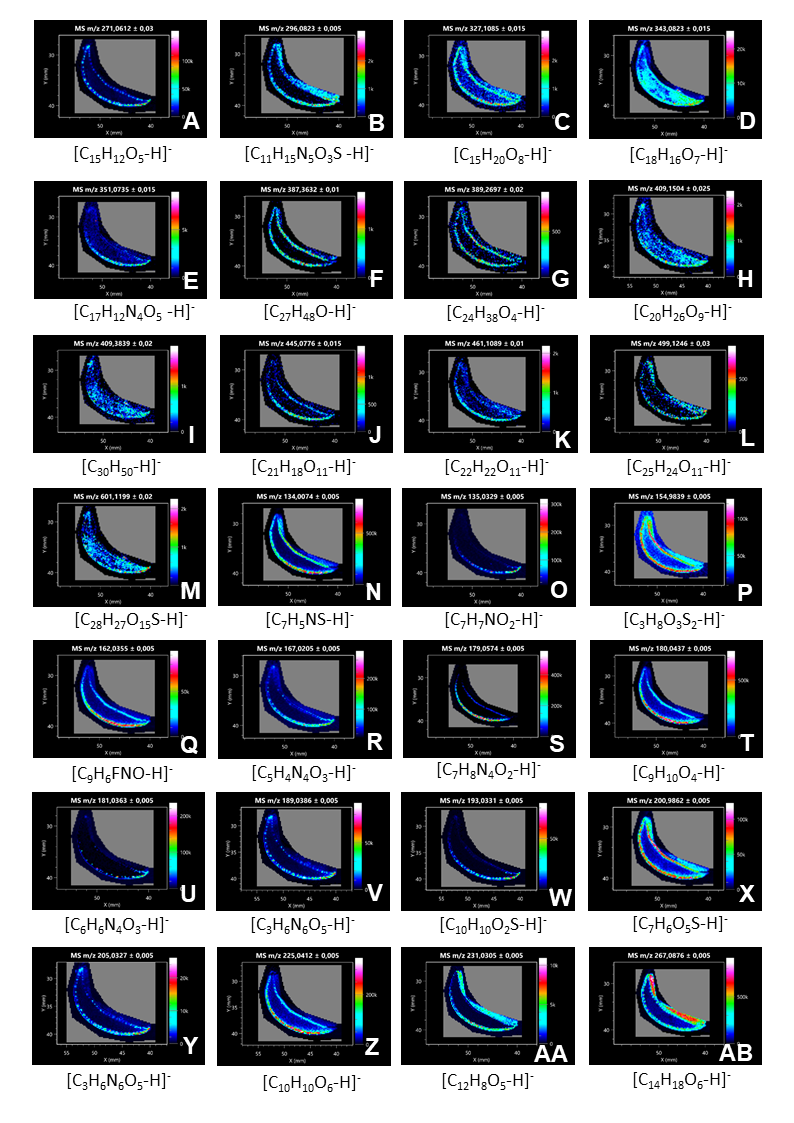


**Fig. S8.** LARAPPI/CI-MSI ion images of compounds identified by UHPLC-UHRMS. **A**-**N** - unidentified, **O** - 4-Aminobenzoate, **P**-**AB** - unidentified.


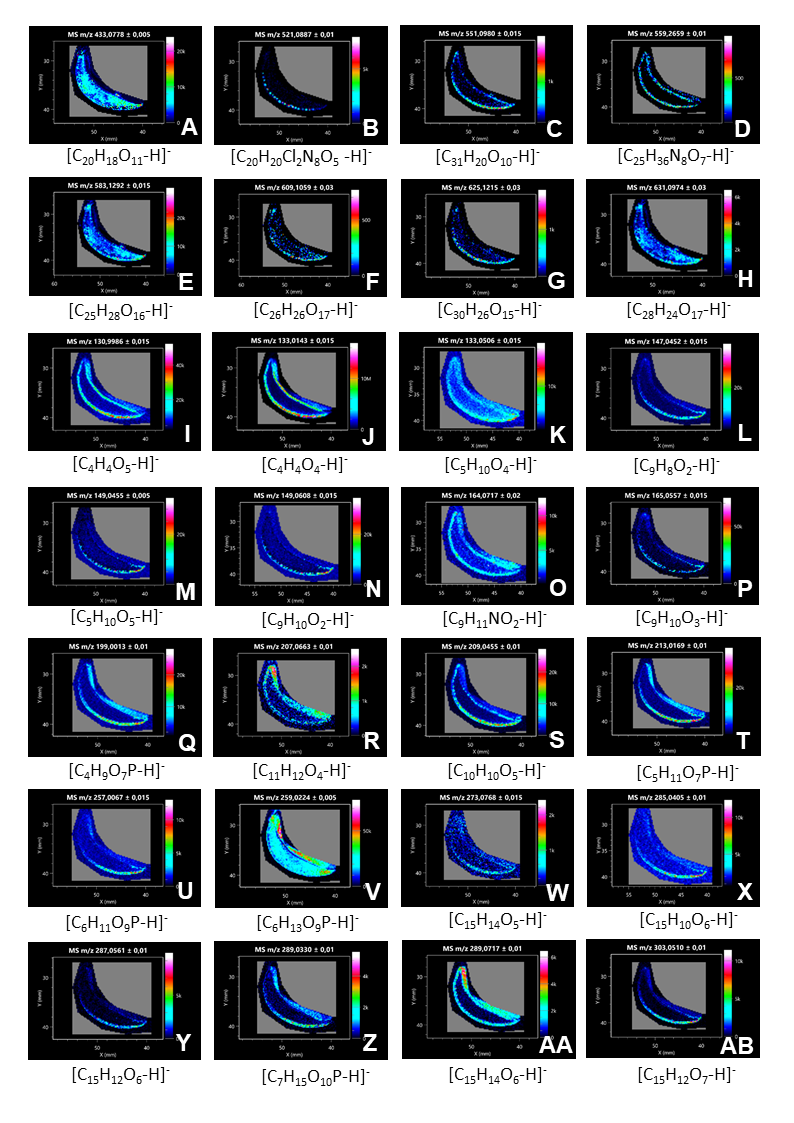


**Fig. S9.** LARAPPI/CI-MSI ion images of compounds identified by UHPLC-UHRMS. **A**-**K** – unidentified, **L** - Cinnamic acid, **M**, **N** - unidentified, **O** - L-Phenylalanine, **P**-**S** - unidentified, **T** - D-Ribose 5-phosphate, **U**, **V**, **W** – unidentified, **X** – Kaempferol, **Y**, **Z**, **AA** – unidentified, **AB** – Quercetin.


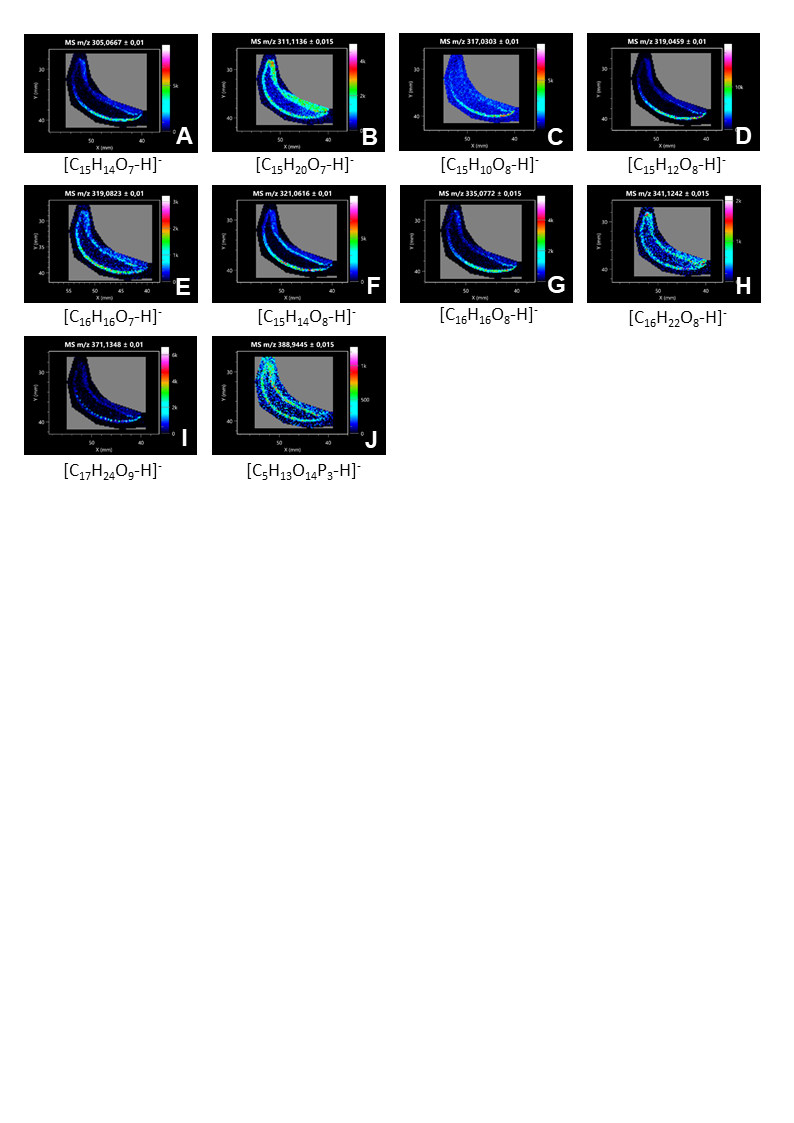


**Fig. S10.** LARAPPI/CI-MSI ion images of compounds identified by UHPLC-UHRMS. **A**-**J** - unidentified.

**Table S1.** Results of UHPLC-HRMS analysis of *Aloe vera* extracts.

| **No.** | **Name** | **Molecular formula** | **Ion polarity** | **Formula of detected ions** | **RT [s]** | ***m/z*_meas._** | **Δ*m/z* [ppm]** | **ΔRT** | **MS/MS score** | **Mean abundance** |
| --- | --- | --- | --- | --- | --- | --- | --- | --- | --- | --- |
| 1 | (R)-3-Hydroxybutyric acid | C_4_H_8_O_3_ | POS | [M+H+CH_3_CN]^+^ | 2.18 | 146.081 | -0.983 | - | - | 10490 |
| 2 | 1,3-Dimethyluric acid | C_7_H_8_N_4_O_3_ | POS | [M+Na]^+^ | 5.44 | 219.0489 | 0.25 | 0.28 | - | 1155 |
| 3 | 1,6-Dimethyl-2(1H)-quinolinone | C_11_H_11_NO | POS | [M+H]^+^ | 6.61 | 174.091 | -1.978 | - | 923.6 | 26463 |
| 4 | 1-Hexadecylamine | C_16_H_35_N | POS | [M+H]^+^ | 12.04 | 242.2837 | -2.084 | - | 817.2 | 13708 |
| 5 | 1-Monolinolenin | C_21_H_36_O_4_ | POS | [M+H]^+^,  [M+H-H_2_O]^+^ | 12.82 | 353.2679 | -2.101 | - | 896 | 60153 |
| 6 | 1-Monolinoleoyl-rac-glycerol | C_21_H_38_O_4_ | POS | [M+H]^+^,  [M+H-H_2_O]^+^ | 13.59 | 355.2833 | -2.897 | - | 859.2 | 16716 |
| 7 | 1-Palmitoyl-2-hydroxy-sn-glycero-3-phosphoethanolamine | C_21_H_44_NO_7_P | POS | [M+H]^+^,  [M+Na]^+^ | 15.68 | 454.2919 | -2.016 | - | 933.5 | 28596 |
| 8 | 13S-Hydroxy-9Z,11E,15Z-octadecatrienoic acid | C_18_H_30_O_3_ | POS | [M+H-H_2_O]^+^, [M+Na]^+^ | 13.78 | 277.2155 | -2.569 | - | 924.5 | 18190 |
| 9 | 2''-O-β-L-galactopyranosylorientin | C_27_H_30_O_16_ | POS | [M+H]^+^ | 6.56 | 611.1599 | -1.206 | - | 809.3 | 24764 |
| 10 | 2-(4-Morpholinyl)benzothiazole | C_11_H_12_N_2_OS | POS | [M+H]^+^ | 10.17 | 221.074 | -1.826 | - | 897.8 | 11202 |
| 11 | 2-Ethyl-2-hydroxybutyric acid | C_6_H_12_O_3_ | POS | [M+H]^+^ | 6.31 | 133.0856 | -2.202 | -0.11 | - | 2924 |
| 12 | 2-Hydroxy-2-methylbutanedioic acid | C_5_H_8_O_5_ | POS | [M+Na]^+^ | 3.38 | 171.0262 | -1.22 | - | - | 928 |
| 13 | 2-Methyl-3-ketovaleric acid | C_6_H_10_O_3_ | POS | [M+NH_4_]^+^ | 1.4 | 148.0966 | -1.193 | - | - | 1397 |
|  |  |  |  | [M+Na]^+^,  [M+H-H_2_O]^+^, [M+H]^+^ | 3.43 | 153.052 | -1.333 | - | - | 44005 |
|  |  |  |  | [M+H+CH_3_CN]^+^ | 5.47 | 172.0964 | -2.185 | - | - | 1160 |
|  |  |  | NEG | [M+OH]^-^ | 4.45 | 147.0663 | 0.002 | - | - | 1401 |
|  |  |  |  | [M-H]^-^ | 5.62 | 129.0556 | -0.653 | - | - | 1177 |
| 14 | 2-Methylquinolin-4-ol | C_10_H_9_NO | POS | [M+H]^+^ | 6.48 | 160.0755 | -1.303 | - | 894.6 | 55468 |
|  |  |  | NEG | [M-H]^-^ | 6.49 | 158.0611 | -0.153 | - | 914.8 | 5057 |
| 15 | 2-Oxoarginine | C_6_H_11_N_3_O_3_ | POS | [M+H]^+^ | 1.67 | 174.087 | -2.007 | - | - | 1703 |
| 16 | 2-Phenylacetamide | C_8_H_9_NO | POS | [M+H]^+^ | 3.06 | 136.0755 | -1.632 | - | 880.4 | 17846 |
| 17 | 3',4'-Methylenedioxy-N-tert-butylcathinone | C_14_H_19_NO_3_ | POS | [M+H]^+^ | 6.39 | 250.1435 | -1.158 | - | 829 | 18578 |
| 18 | 3,4-Dihydroxybenzeneacetic acid | C_8_H_8_O_4_ | POS | [M+NH_4_]^+^ | 5.58 | 186.0758 | -1.317 | -0.15 | - | 2148 |
| 19 | 3,4-Dihydroxyphenylglycol | C_8_H_10_O_4_ | POS | [M+NH_4_]^+^ | 2.24 | 188.0914 | -1.539 | 0.05 | - | 2244. |
| 20 | 3-Aminoisobutanoic acid | C_4_H_9_NO_2_ | POS | [M+H]^+^ | 1.15 | 104.0705 | -0.955 | 0.01 | - | 7536 |
| 21 | 3-Coumaric Acid | C_9_H_8_O_3_ | POS | [M+H-H_2_O]^+^, [M+H]^+^ | 6.4 | 147.0438 | -2.219 | - | 967.2 | 56350 |
| 22 | 3-Hexanone | C_6_H_12_O | POS | [M+NH_4_]^+^ | 0.1 | 118.1227 | 0.764 | - | - | 2385 |
|  |  |  | NEG | [M+HCOO]^-^ | 8.72 | 145.0868 | -1.287 | - | - | 1481 |
| 23 | 3-Hydroxy-4-methoxycinnamic acid | C_10_H_10_O_4_ | POS | [M+H+CH_3_CN]^+^ | 7.09 | 236.0916 | -0.455 | - | - | 3015 |
| 24 | 3-Hydroxycinnamic acid | C_9_H_8_O_3_ | POS | [M+H]^+^,  [M+H-H_2_O]^+^ | 5.89 | 165.0542 | -2.608 | - | 897.1 | 15517 |
| 25 | 3-Hydroxyhippuric acid | C_9_H_9_NO_4_ | POS | [M+H+CH_3_CN]^+^ | 3.76 | 237.0866 | -1.568 | - | - | 2930 |
| 26 | 3-Methoxyphenylacetic acid | C_9_H_10_O_3_ | POS | [M+H]^+^ | 8.81 | 167.07 | -1.902 | 0.06 | - | 5226 |
|  |  |  | NEG | [M-H]^-^ | 4.8 | 117.0558 | 0.711 | 0.11 | - | 985 |
| 27 | 3-Methyladipic acid | C_7_H_12_O_4_ | POS | [M+H+CH_3_CN]^+^ | 6.54 | 202.1072 | -0.822 | 0.04 | - | 1490 |
|  |  |  | NEG | [M-H]^-^ | 6.65 | 159.0659 | -0.898 | 0.15 | - | 1429 |
| 28 | 4',6-Dimethoxyisoflavone-7-O-β-D-glucopyranoside | C_23_H_24_O_10_ | POS | [M+H]^+^ | 7.81 | 461.1434 | -1.835 | - | 905.2 | 5932 |
| 29 | 4-((1E)-3-(2,4-Dihydroxyphenyl)-3-oxoprop-1-en-1-yl)phenyl 6-O-((2E)-3-(4-hydroxyphenyl)prop-2-enoyl)-β-D-glucopyranoside | C_30_H_28_O_11_ | POS | [M+H]^+^ | 8.29 | 565.1694 | -2.184 | - | 966.2 | 6582 |
| 30 | 4-Methylcatechol | C_7_H_8_O_2_ | POS | [M+NH_4_]^+^ | 5.27 | 142.0863 | -0.018 | - | - | 1108 |
|  |  |  |  | [M+H]^+^ | 7.43 | 125.0594 | -2.241 | - | - | 2358 |
| 31 | 4-Pyridoxic acid | C_8_H_9_NO_4_ | POS | [M+H]^+^ | 4.53 | 184.0601 | -1.999 | 0.19 | - | 1951 |
|  |  |  | NEG | [M-H]^-^ | 4.52 | 182.0457 | -0.789 | 0.18 | - | 2700 |
| 32 | 5-Dodecenoic acid | C_12_H_22_O_2_ | POS | [M+H]^+^ | 16.23 | 199.1689 | -2.845 | 0.09 | - | 2800 |
| 33 | 5-Phenylvaleric acid | C_11_H_14_O_2_ | POS | [M+H]^+^ | 11.16 | 179.1061 | -2.654 | -0.06 | - | 2100 |
| 34 | 6-Phosphogluconic acid | C_6_H_13_O_10_P | POS | [M+H]^+^ | 1.23 | 277.0319 | -0.215 | 0.12 | - | 2364 |
| 35 | 7,8-Dihydrobiopterin | C_9_H_13_N_5_O_3_ | POS | [M+NH_4_]^+^ | 7.14 | 257.1353 | -1.332 | - | - | 1312 |
| 36 | 8,11,14-Eicosatrienoic acid | C_20_H_34_O_2_ | POS | [M+H]^+^ | 18.17 | 307.2625 | -2.14 | 0.28 | - | 2853 |
| 37 | 8-[4,5-dihydroxy-6-(hydroxymethyl)-3-[3,4,5-trihydroxy-6-(hydroxymethyl)oxan-2-yl]oxyoxan-2-yl]-5,7-dihydroxy-2-(4-hydroxyphenyl)chromen-4-one | C_27_H_30_O_15_ | POS | [M+H]^+^, [M+Na]^+^ | 6.93 | 595.1649 | -1.184 | - | 929.4 | 460097 |
| 38 | 8-C-Galactosylluteolin | C_21_H_20_O_11_ | POS | [M+H]^+^ | 7.03 | 449.1073 | -1.14 | - | 860 | 9796 |
| 39 | 8Z,11Z,14Z-Eicosatrienoic acid, ethyl ester | C_22_H_38_O_2_ | POS | [M+H]^+^ | 17.13 | 335.2939 | -2.305 | - | 882 | 4260 |
| 40 | 9Z,11E,13E-Octadecatrienoic acid | C_18_H_30_O_2_ | POS | [M+H]^+^, [M+NH_4_]^+^, [M+Na]^+^,  [M+H]-H_2_O]^+^ | 16.85 | 279.2313 | -1.983 | - | 944.4 | 61675 |
| 41 | [(2R,3S,4S,5R,6S)-6-[4-[(E)-3-(2,4-dihydroxyphenyl)-3-oxoprop-1-enyl]phenoxy]-3,4,5-trihydroxyoxan-2-yl]methyl (E)-3-(4-hydroxyphenyl)prop-2-enoate | C_30_H_28_O_11_ | POS | [M+H]^+^,  [M+Na]^+^ | 9.91 | 565.1694 | -1.837 | - | 971 | 69026 |
| 42 | [3,4,5-trihydroxy-6-(hydroxymethyl)oxan-2-yl] 2,4-dihydroxy-6-[(E)-2-phenylethenyl]benzoate | C_21_H_22_O_9_ | POS | [M+H]^+^,  [M+H-H_2_O]^+^ | 7.56 | 419.1328 | -1.989 | - | 848.8 | 18075 |
| 43 | Acetaldehyde | C_2_H_4_O | POS | [M+H+CH_3_CN]^+^ | 1.16 | 86.05992 | -1.36 | - | - | 2181 |
|  |  |  | NEG | [M+HCOO]^-^ | 20.25 | 89.0244 | -0.249 | - | - | 1936 |
| 44 | Acetic acid | C_2_H_4_O_2_ | POS | [M+H+CH_3_CN]^+^ | 1.27 | 102.0548 | -1.234 | - | - | 11386 |
|  |  |  | NEG | [M-H]^-^ | 18.37 | 59.01382 | -0.49 | - | - | 705 |
| 45 | Acetoin | C_4_H_8_O_2_ | POS | [M+H+CH_3_CN]^+^ | 4.91 | 130.0861 | -1.549 | - | - | 3014 |
|  |  |  | NEG | [M+HCOO]^-^ | 2.9 | 133.0506 | 0.078 | - | - | 691 |
| 46 | Acetone | C_3_H_6_O | POS | [M+H+CH_3_CN]^+^ | 5.13 | 100.0755 | -1.488 | - | - | 1447 |
| 47 | Acetylglycine | C_4_H_7_NO_3_ | POS | [M+H+CH_3_CN]^+^ | 1.32 | 159.0763 | -0.798 | -0.03 | - | 3857 |
| 48 | Adenosine 2'-monophosphate | C_10_H_14_N_5_O_7_P | POS | [M+H]^+^ | 2.13 | 348.07 | -1.103 | - | 985.8 | 14605 |
| 49 | Adipic acid | C_6_H_10_O_4_ | POS | [M+H+CH_3_CN]^+^ | 5.46 | 188.0916 | -0.934 | -0.14 | - | 1358 |
| 50 | Alendronate sodium trihydrate | C_4_H_13_NO_7_P_2_ | POS | [M+K]^+^ | 2.21 | 287.9799 | -0.167 | - | - | 3763 |
| 51 | Allantoin | C_4_H_6_N_4_O_3_ | POS | [M+Na]^+^ | 1.22 | 181.0333 | 0.312 | - | - | 1551 |
|  |  |  | NEG | [M-H]^-^ | 1.12 | 157.037 | 2.224 | - | - | 3548 |
| 52 | Aloeresin A | C_28_H_28_O_11_ | POS | [M+H]^+^ | 7.87 | 541.1695 | -1.786 | - | 961.4 | 52969 |
|  |  |  | NEG | [M-H]^-^ | 7.87 | 539.155 | -1.736 | - | 889.6 | 26626 |
| 53 | Aloeresin D | C_29_H_32_O_11_ | POS | [M+H]^+^, [M+Na]^+^ | 8.38 | 557.2004 | -2.463 | - | 874.5 | 1275492 |
| 54 | α-Terpineol | C_10_H_18_O | POS | [M+NH_4_]^+^ | 11.71 | 172.1692 | -0.301 | -0.06 | - | 1204 |
| 55 | Aniline | C_6_H_7_N | POS | [M+H]^+^ | 20.51 | 94.06487 | -2.684 | - | 808.3 | 20948 |
|  |  |  | NEG | [M+HCOO]^-^ | 7.4 | 138.0558 | -1.805 | -0.03 | - | 1312 |
| 56 | Arachidic acid | C_20_H_40_O_2_ | POS | [M+H+CH_3_CN]^+^ | 17.8 | 354.3365 | -0.581 | - | - | 16975 |
| 57 | Astragalin | C_21_H_20_O_11_ | POS | [M+H]^+^, [M+Na]^+^ | 7.94 | 449.107 | -1.883 | - | 974.9 | 32515 |
| 58 | Azelaic acid | C_9_H_16_O_4_ | POS | [M+H-H_2_O]^+^, [M+Na]^+^, [M+H]^+^ | 8.41 | 171.1012 | -2.419 | 0.14 | - | 3370 |
|  |  |  | NEG | [M-H]^-^ | 8.41 | 187.0973 | -1.462 | 0.15 | - | 10723 |
| 59 | Behenic acid | C_22_H_44_O_2_ | POS | [M+NH_4_]^+^ | 15.5 | 358.367 | -2.575 | - | - | 306688 |
| 60 | Benzaldehyde | C_7_H_6_O | POS | [M+NH_4_]^+^ | 7.1 | 124.0755 | -1.901 | - | - | 1296 |
| 61 | Benzoic acid | C_7_H_6_O_2_ | POS | [M+H]^+^ | 8.55 | 123.0437 | -2.941 | 0.11 | - | 4830 |
|  |  |  | NEG | [M+H_2_O-H]^-^ | 6.32 | 138.0561 | 0.252 | - | 0.02 | 1897 |
| 62 | β-N-Acetylglucosamine | C_8_H_15_NO_6_ | POS | [M+K]^+^ | 1.17 | 260.0529 | -0.849 | 0.03 | - | 2692 |
| 63 | Biotin | C_10_H_16_N_2_O_3_S | POS | [M+H]^+^ | 5.21 | 245.0956 | 0.832 | - | - | 1365 |
| 64 | Butanal | C_4_H_8_O | POS | [M+H]^+^ | 7.88 | 73.06461 | -2.508 | - | - | 2250 |
|  |  |  |  | [M+H+CH_3_CN]^+^ | 20.92 | 114.0911 | -2.354 | - | - | 820 |
| 65 | Butyl 4-hydroxybenzoate | C_11_H_14_O_3_ | POS | [M+H]^+^ | 13.22 | 195.1011 | -2.323 | - | 969.8 | 10378 |
| 66 | Cellobiose | C_12_H_22_O_11_ | POS | [M+K]^+^ | 1.17 | 381.079 | -0.976 | -0.04 | - | 572999 |
|  |  |  |  | [M+Na]^+^ | 1.17 | 365.1051 | -0.788 | -0.04 | - | 141549 |
| 67 | Chelidonic acid | C_7_H_4_O_6_ | POS | [M+H]^+^ | 2.14 | 185.0079 | -1.141 | - | 984.4 | 64776 |
|  |  |  | NEG | [M-H]^-^, [2M-H]^-^ | 2.12 | 182.9933 | -0.875 | - | 996.3 | 32821 |
| 68 | Chlorogenic acid | C_16_H_18_O_9_ | POS | [M+H]^+^ | 6.23 | 355.1019 | -1.264 | 0.09 | - | 13000 |
|  |  |  |  | [M+Na]^+^ | 6.34 | 377.0844 | 0.571 | 0.2 | - | 2183 |
|  |  |  | NEG | [M-H]^-^ | 6.23 | 353.0877 | -0.358 | 0.09 | - | 11207 |
| 69 | Chromomoric acid B | C_18_H_28_O_3_ | POS | [M+H]^+^,  [M+H-H_2_O]^+^ | 9.95 | 293.2104 | -2.273 | - | 869.3 | 17217 |
|  |  |  |  | [M+H]^+^, [M+Na]^+^ | 13.38 | 293.2104 | -1.87 | - | 921.5 | 43213 |
| 70 | Citric acid | C_6_H_8_O_7_ | POS | [M+N]a^+^ | 1.75 | 215.0161 | -0.667 | -0.29 | - | 46054 |
|  |  |  |  | [M+Na]^+^,  [M+H-H_2_O]^+^, [M+NH_4_]^+^, [M+H]^+^ | 2.21 | 215.0161 | -0.783 | 0.18 | - | 77983 |
|  |  |  | NEG | [M-H]^-^ | 2.2 | 191.0198 | 0.278 | 0.16 | - | 481849 |
| 71 | Citrulline | C_6_H_13_N_3_O_3_ | POS | [M+H]^+^ | 1.33 | 176.1029 | -0.662 | 0.28 | - | 1285 |
| 72 | CocamidopropylBetaine | C_19_H_38_N_2_O_3_ | POS | [M+H]^+^ | 11.95 | 343.2949 | -1.705 | - | 990.2 | 5165 |
| 73 | Coumarin | C_9_H_6_O_2_ | POS | [M+H]^+^ | 6.77 | 147.0438 | -1.601 | - | 902.4 | 27394 |
|  |  |  | NEG | [M+HCOO]^-^ | 8.99 | 191.0348 | -0.209 | -0.03 | - | 14180 |
| 74 | Cyclohexanone | C_6_H_10_O | POS | [M+H]^+^ | 3.55 | 99.08028 | -1.65 | - | - | 920 |
| 75 | Cytidine | C_9_H_13_N_3_O_5_ | POS | [M+Na+CH_3_CN]^+^ | 1.16 | 307.1007 | -2.079 | -0.22 | - | 1364 |
| 76 | D-Fructose | C_6_H_12_O_6_ | POS | [M+K]^+^ | 1.12 | 219.0265 | -0.453 | 0 | - | 25547 |
|  |  |  |  | [M+Na]^+^ | 1.24 | 203.0526 | -0.242 | 0.12 | - | 14584 |
|  |  |  |  | [M+NH_4_]^+^ | 1.37 | 198.097 | -1.334 | 0.25 | - | 1514 |
| 77 | D-Limonene | C_10_H_16_ | POS | [M+NH_4_]^+^ | 7.32 | 154.1587 | -2.099 | - | - | 1058 |
| 78 | D-Ribose 5-phosphate | C_5_H_11_O_8_P | POS | [M+K]^+^ | 1.14 | 268.9824 | -0.264 | 0.11 | - | 3568 |
|  |  |  |  | [M+Na]^+^ | 1.15 | 253.0081 | -1.356 | 0.12 | - | 3118 |
| 79 | D-Serine | C_3_H_7_NO_3_ | POS | [M+H]^+^ | 1.28 | 106.0498 | -0.918 | -0.06 | - | 6514 |
| 80 | D-Threo-isocitric acid | C_6_H_8_O_7_ | POS | [M+K]^+^ | 1.19 | 230.99 | -0.672 | -0.13 | - | 59178 |
|  |  |  |  | [M+Na]^+^ | 1.19 | 215.0161 | -0.494 | -0.13 | - | 50332 |
|  |  |  |  | [M+H]^+^ | 1.7 | 193.0342 | -0.463 | - | 933.5 | 17187 |
| 81 | Decanal | C_10_H_20_O | POS | [M+H+CH_3_CN]^+^ | 18.33 | 198.1849 | -1.931 | - | - | 3685 |
| 82 | Dehydroascorbic acid | C_6_H_6_O_6_ | POS | [M+Na]^+^ | 1.2 | 197.0057 | 0.225 | -0.11 | - | 13994 |
|  |  |  | NEG | [M-H]^-^,  [M-H-H_2_O]^-^ | 1.18 | 173.0092 | 0.543 | -0.13 | - | 420039 |
|  |  |  |  | [M+H_2_O-H]^-^ | 1.28 | 191.0198 | 0.397 | -0.03 | - | 565778 |
| 83 | Deoxyguanosine | C_10_H_13_N_5_O_4_ | POS | [M+H]^+^ | 4.44 | 268.1037 | -1.196 | -0.12 | - | 35460 |
| 84 | Diethanolamine | C_4_H_11_NO_2_ | POS | [M+K+CH_3_CN]^+^ | 1.07 | 185.0687 | 0.268 | 0.09 | - | 1028 |
| 85 | Dilinolenin (9c,12c,15c) | C_39_H_64_O_5_ | POS | [M+H]^+^ | 16.15 | 613.4817 | -1.511 | - | 911.5 | 13758 |
| 86 | Dimethylmalonic acid | C_5_H_8_O_4_ | POS | [M+H]^+^ | 5.47 | 133.0493 | -1.589 | 0.01 | - | 1180 |
| 87 | Dodecanedioic acid | C_12_H_22_O_4_ | POS | [M+Na]^+^ | 10.33 | 253.1408 | -0.931 | -0.29 | - | 1187 |
|  |  |  | NEG | [M-H]^-^ | 10.78 | 229.1441 | -2.079 | 0.16 | - | 1389 |
| 88 | Dopamine | C_8_H_11_NO_2_ | POS | [M+H]^+^ | 6.77 | 154.0861 | -1.275 | - | - | 17860 |
| 89 | Erucamide | C_22_H_43_NO | POS | [M+H]^+^ | 14.12 | 338.3409 | -1.875 | - | 962.4 | 52880 |
| 90 | Erythrose | C_4_H_8_O_4_ | POS | [M+H+CH_3_CN]^+^ | 1.76 | 162.0759 | -1.321 | - | - | 1643 |
| 91 | Ethyl isopropyl ketone | C_6_H_12_O | POS | [M+H+CH_3_CN]^+^ | 5.11 | 142.1223 | -2.187 | - | - | 869 |
|  |  |  | NEG | [M+HCOO]^-^ | 9.2 | 145.087 | 0.155 | - | - | 1361 |
| 92 | Eucalyptol | C_10_H_18_O | POS | [M+NH_4_]^+^ | 6.29 | 172.1694 | -1.37 | - | - | 2808 |
|  |  |  |  | [M+H]^+^ | 8.85 | 155.1428 | -1.404 | - | - | 1797 |
|  |  |  | NEG | [M+H]COO^-^ | 9.93 | 199.1335 | -2.388 | - | - | 1989 |
| 93 | Flavone base + 4O, O-MalonylHex | C_24_H_22_O_14_ | POS | [M+H]^+^ | 8.2 | 535.1074 | -1.451 | - | 967.6 | 20811 |
| 94 | Formaldehyde | CH_2_O | POS | [M+H+CH_3_CN]^+^ | 4.89 | 72.04439 | 0.045 | - | - | 2304 |
| 95 | Formamide | CH_3_NO | POS | [M+H+CH_3_CN]^+^ | 1.26 | 87.05512 | -1.914 | - | - | 5314 |
| 96 | γ-Tocopherol | C_28_H_48_O_2_ | POS | [M+H]^+^ | 16.79 | 417.3717 | -2.497 | - | - | 4348 |
| 97 | Genistein | C_15_H_10_O_5_ | POS | [M+H]^+^ | 9.95 | 271.0596 | -1.892 | 0.11 | - | 2368 |
|  |  |  | NEG | [M-H]^-^ | 9.95 | 269.045 | -1.651 | 0.11 | - | 2261 |
| 98 | Glutathione | C_10_H_17_N_3_O_6_S | POS | [M+Na+CH_3_CN]^+^ | 16.72 | 371.1002 | 1.437 | - | - | 2092 |
|  |  |  |  | [M+K]^+^ | 1.21 | 346.0465 | -1.476 | - | - | 1360 |
|  |  |  |  | [M+H]^+^ | 2.15 | 308.0908 | -1.057 | - | - | 86928 |
|  |  |  | NEG | [M-H]^-^ | 2.13 | 306.0764 | -0.559 | - | - | 9825 |
| 99 | Glycerol | C_3_H_8_O_3_ | POS | [M+H+CH_3_CN]^+^ | 1.32 | 134.081 | -1.193 | 0.2 | - | 1008 |
| 100 | Glycyl-L-leucine | C_8_H_16_N_2_O_3_ | POS | [M+H]^+^ | 5.48 | 189.1231 | -1.484 | 0.16 | - | 1373 |
| 101 | Glycylproline | C_7_H_12_N_2_O_3_ | POS | [M+H]^+^ | 1.32 | 173.092 | -0.645 | -0.02 | - | 2762 |
| 102 | Guanosine | C_10_H_13_N_5_O_5_ | POS | [M+H]^+^ | 4.62 | 284.0986 | -1.385 | 0.18 | - | 6516 |
|  |  |  | NEG | [M-H]^-^ | 4.61 | 282.0843 | -0.515 | 0.17 | - | 2773 |
| 103 | Heneicosanoic acid | C_21_H_42_O_2_ | POS | [M+K+CH_3_CN]^+^ | 15.3 | 406.307 | -2.934 | - | - | 7307 |
| 104 | Heptadecanoic acid | C_17_H_34_O_2_ | POS | [M+NH_4_]^+^ | 11.63 | 288.2891 | -2.17 | - | - | 29638 |
| 105 | Histamine | C_5_H_9_N_3_ | POS | [M+H]^+^ | 0.98 | 112.0869 | 0.111 | 0.13 | - | 2500 |
| 106 | Homo-L-arginine | C_7_H_16_N_4_O_2_ | POS | [M+H]^+^ | 1.15 | 189.1343 | -1.937 | 0.16 | - | 7375 |
| 107 | Homovanillic acid | C_9_H_10_O_4_ | POS | [M+NH_4_]^+^ | 6.98 | 200.0914 | -1.612 | 0.19 | - | 2551 |
| 108 | Hydroxyindoleacetic acid | C_10_H_9_NO_3_ | POS | [M+H+CH_3_CN]^+^ | 2.66 | 233.0919 | -0.988 | - | - | 5014 |
| 109 | Imidazole | C_3_H_4_N_2_ | POS | [M+H+CH_3_CN]^+^ | 1.17 | 110.0712 | -0.64 | 0.12 | - | 2104 |
|  |  |  | NEG | [M+HCOO]^-^ | 1.05 | 113.0356 | -0.126 | 0 | - | 1636 |
| 110 | Indole-3-butyric acid | C_12_H_13_NO_2_ | POS | [M+H]^+^ | 6.47 | 204.1018 | -0.735 | - | 845.6 | 10279 |
| 111 | Isobutyric acid | C_4_H_8_O_2_ | POS | [M+H+CH_3_CN]^+^ | 6.24 | 130.0861 | -1.467 | -0.13 | - | 1784 |
| 112 | Isoguanine | C_5_H_5_N_5_O | POS | [M+H]^+^ | 4.61 | 152.0564 | -1.69 | - | - | 6907 |
| 113 | Isopropyl alcohol | C_3_H_8_O | POS | [M+H+CH_3_CN]^+^ | 0.32 | 102.0912 | -1.568 | - | - | 1495 |
| 114 | Isovalerylglycine | C_7_H_13_NO_3_ | POS | [M+H+CH_3_CN]^+^ | 6.14 | 201.123 | -2.158 | 0.14 | - | 4204 |
|  |  |  | NEG | [M-H]^-^ | 5.92 | 158.0822 | -0.63 | -0.09 | - | 14810 |
| 115 | Kaempferol | C_15_H_10_O_6_ | POS | [M+H]^+^ | 10.08 | 287.0546 | -1.63 | 0.14 | - | 3678 |
|  |  |  | NEG | [M-H]^-^ | 10.1 | 285.04 | -1.055 | 0.15 | - | 7926 |
| 116 | Kaempferol-3-*O*-rutinoside | C_27_H_30_O_15_ | POS | [M+H]^+^, [M+Na]^+^ | 7.72 | 595.1648 | -1.636 | - | 990.4 | 199990 |
|  |  |  | NEG | [M-H]^-^ | 7.61 | 593.1505 | -1.292 | - | 958.8 | 57485 |
| 117 | L-3-Phenyllactic acid | C_9_H_10_O_3_ | POS | [M+H]^+^ | 7.61 | 167.07 | -1.779 | 0.01 | - | 3100 |
|  |  |  | NEG | [M-H]^-^ | 7.61 | 165.0557 | -0.212 | 0.01 | - | 1119 |
| 118 | L-Arginine | C_6_H_14_N_4_O_2_ | POS | [M+H]^+^ | 1.16 | 175.1188 | -0.809 | 0.22 | - | 233006 |
| 119 | L-Asparagine | C_4_H_8_N_2_O_3_ | POS | [M+K]^+^ | 1.14 | 171.0167 | 0.066 | 0.14 | - | 3909 |
|  |  |  | NEG | [M-H]^-^ | 1.04 | 131.0463 | 0.956 | 0.04 | - | 8937 |
| 120 | L-Aspartic acid | C_4_H_7_NO_4_ | POS | [M+H]^+^,  [M+H-CO_2_]^+^ | 1.31 | 134.0447 | -0.891 | 0.26 | - | 6296 |
|  |  |  |  | [M+H+CH_3_CN]^+^ | 1.31 | 175.071 | -2.044 | 0.25 | - | 1303 |
| 121 | L-Glutamic acid | C_5_H_9_NO_4_ | POS | [M+H]^+^ | 1.31 | 148.0603 | -0.787 | -0.04 | - | 35982 |
| 122 | L-Glutamine | C_5_H_10_N_2_O_3_ | POS | [M+K]^+^ | 1.14 | 185.0323 | -0.174 | 0.12 | - | 1729 |
|  |  |  | NEG | [M-H]^-^ | 1.08 | 145.0619 | 0.464 | 0.07 | - | 2685 |
| 123 | L-Isoleucine | C_6_H_13_NO_2_ | POS |  |  |  |  |  |  |  |
|  |  |  |  | [M+H]^+^ | 2.92 | 132.1016 | -1.974 | 0.12 | - | 327902 |
|  |  |  | NEG | [M-H]^-^ | 2.89 | 130.0873 | -0.612 | 0.09 | - | 29153 |
| 124 | L-Kynurenine | C_10_H_12_N_2_O_3_ | POS | [M+H]^+^ | 4.9 | 209.0918 | -0.868 | 0.11 | - | 5096 |
| 125 | L-Lactic acid | C_3_H_6_O_3_ | POS | [M+H+CH_3_CN]^+^ | 1.27 | 132.0654 | -0.654 | -0.1 | - | 2107 |
| 126 | L-Lysine | C_6_H_14_N_2_O_2_ | POS | [M+H]^+^ | 0.93 | 147.1127 | -0.736 | 0.02 | - | 11649 |
|  |  |  | NEG | [M-H]^-^ | 0.92 | 145.0984 | 1.048 | 0.02 | - | 1353 |
| 127 | L-Malic acid | C_4_H_6_O_5_ | POS | [M+NH_4_]^+^ | 1.58 | 152.055 | -2.341 | 0.28 | - | 1490 |
| 128 | L-Phenylalanine | C_9_H_11_NO_2_ | POS | [M+H]^+^,  [M+H-H_2_O]^+^ | 5.02 | 166.0858 | -2.632 | 0.12 | - | 1828113 |
|  |  |  | NEG | [M-H]^-^, [2M-H]^-^ | 5.03 | 164.0716 | -0.747 | 0.13 | - | 297328 |
| 129 | L-Proline | C_5_H_9_NO_2_ | POS | [M+H]^+^ | 1.17 | 116.0706 | -0.433 | 0.05 | - | 57320 |
| 130 | L-Thyronine | C_15_H_15_NO_4_ | POS | [M+H+CH_3_CN]^+^ | 7.19 | 315.1336 | -1.201 | 0.28 | - | 1156 |
| 131 | L-Tryptophan | C_11_H_12_N_2_O_2_ | POS | [M+Na]^+^ | 6 | 227.0784 | -2.971 | 0.2 | - | 2782 |
|  |  |  | NEG | [M-H]^-^, [2M-H]^-^ | 5.98 | 203.0824 | -0.997 | 0.18 | - | 473721 |
| 132 | Leu-Leu | C_12_H_24_N_2_O_3_ | POS | [M+H]^+^ | 6.9 | 245.1858 | -0.57 | - | 930.6 | 8695 |
| 133 | Levoglucosan | C_6_H_10_O_5_ | POS | [M+H+CH_3_CN]^+^ | 1.37 | 204.0864 | -1.139 | - | - | 3866 |
|  |  |  |  | [M+H]^+^,  [M+H-H_2_O]^+^ | 1.36 | 163.06 | -0.863 | - | - | 19094 |
|  |  |  |  | [M-H]^-^ | 1.07 | 161.0456 | 0.594 | - | - | 1586 |
| 134 | Lidocaine | C_14_H_22_N_2_O | POS | [M+H]^+^ | 7.12 | 235.1798 | -2.995 | - | 963.1 | 8061 |
| 135 | LPC 18:2 | C_26_H_50_NO_7_P | POS | [M+H]^+^ | 15.83 | 520.3387 | -2.06 | - | 968.5 | 18447 |
| 136 | LPE 18:2^a^ | C_23_H_44_NO_7_P | POS | [M+H]^+^, [M+Na]^+^ | 14.2 | 478.2918 | -2.189 | - | 932.4 | 63830 |
| 137 | LPE 18:3^a^ | C_23_H_42_NO_7_P | POS | [M+H]^+^, [M+Na]^+^ | 13.28 | 476.2761 | -2.329 | - | 847.4 | 40656 |
| 138 | Malic acid | C_4_H_6_O_5_ | POS | [M+H]^+^ | 1.58 | 135.0287 | -0.913 | 0.28 | - | 7435 |
|  |  |  | NEG | [M-H]^-^,  [M-H-H_2_O]^-^ | 1.15 | 133.0143 | 0.169 | -0.15 | - | 1094075 |
|  |  |  |  | [M-H]^-^ | 2.39 | 133.0142 | -0.274 | - | 894.4 | 14865 |
| 139 | Mannitol | C_6_H_14_O_6_ | POS | [M+K]^+^ | 1.12 | 221.0417 | -2.066 | 0.08 | - | 1059 |
| 140 | Mannose 6-phosphate | C_6_H_13_O_9_P | POS | [M+H]^+^ | 1.32 | 261.0368 | -0.792 | 0.23 | - | 2423 |
| 141 | Menthol | C_10_H_20_O | POS | [M+H+CH_3_CN]^+^ | 11.96 | 198.1849 | -1.739 | - | - | 2590 |
| 142 | Methionine sulfoxide | C_5_H_11_NO_3_S | POS | [M+H]^+^ | 1.33 | 166.0531 | -0.498 | 0.29 | - | 5852 |
| 143 | Methylnissolin-3-O-glucoside | C_23_H_26_O_10_ | POS | [M+H]^+^,  [M+H-H_2_O]^+^, [M+NH_4_]^+^ | 7.19 | 463.1592 | -1.546 | - | 834.4 | 23560 |
| 144 | Methylsuccinic acid | C_5_H_8_O_4_ | POS | [M+H+CH_3_CN]^+^ | 1.79 | 174.0761 | -0.08 | - | - | 1472 |
| 145 | N-Acetyl-D-glucosamine | C_8_H_15_NO_6_ | POS | [M+H+CH_3_CN]^+^ | 1.18 | 263.1237 | -0.286 | 0.04 | - | 1601 |
|  |  |  |  | [M+H]^+^ | 1.38 | 222.0971 | -0.592 | 0.25 | - | 1702 |
| 146 | N-Acetylputrescine | C_6_H_14_N_2_O | POS | [M+H]^+^ | 1.19 | 131.1178 | -0.88 | -0.05 | - | 1492 |
| 147 | N6-Succinyladenosine | C_14_H_17_N_5_O_8_ | POS | [M+H]^+^ | 5.42 | 384.1144 | -1.573 | - | 957.5 | 27862 |
| 148 | Naringin | C_27_H_32_O_14_ | POS | [M+H]^+^ | 7.69 | 581.1877 | 2.957 | -0.1 | - | 1379 |
|  |  |  | NEG | [M-H]^-^ | 7.56 | 579.171 | -1.633 | -0.23 | - | 11307 |
|  |  |  |  | [M+HCOO]^-^ | 7.66 | 625.1765 | -1.542 | -0.13 | - | 10019 |
|  |  |  |  | [M+HCOO]^-^, [M+Cl]^-^ | 7.99 | 625.1764 | -1.558 | 0.2 | - | 6907 |
| 149 | 7-Hydroxy-5-methyl-2-(2-oxopropyl)-8-[3,4,5-trihydroxy-6-(hydroxymethyl)oxan-2-yl]chromen-4-one | C_19_H_22_O_9_ | POS | [M+H]^+^, [M+Na]^+^, [M+K]^+^ | 6.19 | 395.133 | -1.77 | - | 839.3 | 1763141 |
| 150 | 7-Hydroxy-3-(2-hydroxypropyl)-5-methylisochromen-1-one | C_13_H_14_O_4_ | POS | [M+H]^+^ | 8.06 | 235.096 | -1.97 | - | 929.1 | 7120 |
| 151 | (1S,3R,4S,5R)-1,3,5-Trihydroxy-4-{[(2E)-3-(4-hydroxyphenyl)-2-propenoyl]oxy}cyclohexanecarboxylic acid | C_16_H_18_O_8_ | POS | [M+H]^+^, [M+Na]^+^ | 6.26 | 339.107 | -1.206 | - | 925.3 | 17142 |
| 152 | (1S,3R,4R,5R)-1,3,4-Trihydroxy-5-[(E)-3-(4-hydroxyphenyl)prop-2-enoyl]oxycyclohexane-1-carboxylic acid | C_16_H_18_O_8_ | POS | [M+H]^+^,  [M+Na]^+^, [M+K]^+^,  [M+H-H_2_O]^+^ | 6.88 | 339.107 | -1.466 | - | 931.8 | 211836 |
| 153 | 6-[(2S,3R,4S,5S,6R)-4,5-Dihydroxy-6-(hydroxymethyl)-3-[(2S,3R,4S,5S)-3,4,5-trihydroxyoxan-2-yl]oxyoxan-2-yl]-5,7-dihydroxy-2-(4-hydroxyphenyl)chromen-4-one | C_26_H_28_O_14_ | POS | [M+H]^+^ | 7.27 | 565.1543 | -1.506 | - | 935.7 | 32223 |
| 154 | 4-(2,6,6-Trimethyl-4-oxo-2-cyclohexen-1-yl)-2-butanyl beta-D-glucopyranoside | C_19_H_32_O_7_ | POS | [M+H]^+^, [M+Na]^+^ | 8.13 | 373.2213 | -2.728 | - | 908.5 | 16303 |
| 155 | 1,8,10-trihydroxy-3-(hydroxymethyl)-10-[3,4,5-trihydroxy-6-(hydroxymethyl)oxan-2-yl]anthracen-9-one | C_21_H_22_O_10_ | POS | [M+H]^+^ | 6.36 | 435.128 | -1.278 | - | 818 | 9238 |
|  |  |  | NEG | [M+HCOO]^-^, [M+Cl]^-^ | 7.84 | 479.1188 | -1.536 | - | 946.6 | 10713 |
| 156 | 5,7-Dihydroxy-3-(4-hydroxyphenyl)-6,8-bis[3,4,5-trihydroxy-6-(hydroxymethyl)oxan-2-yl]chromen-4-one | C_27_H_30_O_15_ | POS | [M+H]^+^, [M+Na]^+^ | 6.63 | 595.1649 | -1.391 | - | 850.8 | 74264 |
| 157 | Nonadecanoic acid | C_19_H_38_O_2_ | POS | [M+NH_4_]^+^ | 13.26 | 316.3202 | -2.075 | - | - | 191528 |
| 158 | Norepinephrine | C_8_H_11_NO_3_ | POS | [M+NH_4_]^+^ | 1.28 | 187.1078 | 0.215 | 0.15 | - | 1297 |
| 159 | o-Cresol | C_7_H_8_O | POS | [M+H]^+^ | 9.54 | 109.0646 | -2.247 | 0.09 | - | 1780 |
| 160 | Octadecanol | C_18_H_38_O | POS | [M+H+CH_3_CN]^+^ | 17.22 | 312.3255 | -1.955 | - | - | 2428 |
| 161 | Octanal | C_8_H_16_O | POS | [M+H]^+^ | 12.85 | 129.1271 | -2.236 | - | - | 1376 |
|  |  |  | NEG | [M+HCOO]^-^ | 10.3 | 173.1181 | -1.296 | - | - | 7333 |
| 162 | Oleamide | C_18_H_35_NO | POS | [M+H]^+^, [M+Na]^+^ | 17.96 | 282.2784 | -2.547 | - | 948.1 | 586660 |
|  |  |  |  | [M+H]^+^ | 18.21 | 282.2786 | -1.005 | - | 827.6 | 52449 |
| 163 | Oleic acid | C_18_H_34_O_2_ | POS | [M+Na]^+^ | 17.23 | 305.2461 | 2.664 | - | - | 1551 |
| 164 | Oxoglutaric acid | C_5_H_6_O_5_ | POS | [M+H]^+^ | 1.66 | 147.0287 | -0.992 | 0.21 | - | 20764 |
|  |  |  | NEG | [M-H]^-^ | 1.07 | 131.0826 | 0.264 | 0.15 | - | 1489 |
| 165 | p-Aminobenzoic acid | C_7_H_7_NO_2_ | POS | [M+H]^+^ | 5.45 | 138.055 | 0.388 | 0.24 | - | 1627 |
| 166 | p-Cresol | C_7_H_8_O | POS | [M+NH_4_]^+^ | 7.3 | 126.0911 | -2.006 | -0.14 | - | 1716 |
| 167 | p-Hydroxyphenylacetic acid | C_8_H_8_O_3_ | POS | [M+H]^+^,  [M+H-H_2_O]^+^ | 6.46 | 153.0542 | -2.475 | -0.02 | - | 10923 |
|  |  |  | NEG | [M-H]^-^ | 6.45 | 151.0401 | 0.489 | -0.02 | - | 1339 |
| 168 | p-Octopamine | C_8_H_11_NO_2_ | POS | [M+H]^+^ | 6.26 | 154.0862 | -0.132 | - | - | 2838 |
|  |  |  | NEG | [M-H]^-^ | 6.78 | 152.0716 | -0.829 | - | - | 2029 |
| 169 | Pantothenic acid | C_9_H_17_NO_5_ | POS | [M+H+CH_3_CN]^+^ | 4.45 | 261.1444 | -0.323 | - | - | 1170 |
|  |  |  |  | [M+H]^+^,  [M+H-H_2_O]^+^, [M+Na]^+^ | 5.34 | 220.1177 | -1.208 | - | - | 101534 |
|  |  |  |  | [M+H+CH_3_CN]^+^, [M+Na-H_2_O]^+^ | 6.07 | 261.1441 | -0.661 | - | - | 1905 |
|  |  |  | NEG | [M-H]^-^, [2M-H]^-^ | 5.34 | 218.1033 | -0.619 | - | - | 65764 |
| 170 | PC O-18:1 | C_26_H_52_NO_7_P | POS | [M+H]^+^ | 13.7 | 522.3542 | -1.589 | - | 964.2 | 2442 |
| 171 | PE O-15:1_3:0 | C_23_H_46_NO_7_P | POS | [M+H]^+^ | 15.57 | 480.3073 | -2.724 | - | 876 | 7981 |
| 172 | Phenylacetaldehyde | C_8_H_8_O | POS | [M+H]^+^ | 5.04 | 121.0645 | -2.44 | -0.19 | - | 1402 |
| 173 | Phenylpropiolic acid | C_9_H_6_O_2_ | POS | [M+H+CH_3_CN]^+^ | 8.69 | 188.0701 | -2.644 | -0.2 | - | 1964 |
| 174 | Pheophorbide A | C_35_H_36_N_4_O_5_ | POS | [M+H]^+^, [M+Na]^+^ | 18 | 593.2745 | -2.223 | - | 941.7 | 1508459 |
|  |  |  |  | [M+H]^+^ | 18.26 | 593.2747 | -2.007 | - | 846 | 26626 |
| 175 | Phthalic acid | C_8_H_6_O_4_ | POS | [M+H-H_2_O]^+^, [M+H]^+^ | 6.67 | 149.023 | -2.139 | 0.1 | - | 10713 |
|  |  |  | NEG | [M-H]^-^ | 6.67 | 165.0191 | -1.134 | 0.11 | - | 17766 |
| 176 | Pipecolic acid | C_6_H_11_NO_2_ | POS | [M+H]^+^ | 1.18 | 130.0861 | -1.17 | -0.16 | - | 12763 |
| 177 | Propyl alcohol | C_3_H_8_O | POS | [M+H]^+^ | 0.22 | 61.06493 | 1.857 | - | - | 4045 |
| 178 | Propylene glycol | C_3_H_8_O_2_ | POS | [M+H+CH_3_CN]^+^ | 1.45 | 118.0861 | -1.233 | 0.09 | - | 146048 |
| 179 | Pyridoxine | C_8_H_11_NO_3_ | POS | [M+H]^+^ | 2.28 | 170.0809 | -1.529 | - | - | 2672 |
|  |  |  | NEG | [M-H]^-^ | 4.94 | 168.0664 | -1.311 | - | - | 5235 |
| 180 | Pyrocatechol | C_6_H_6_O_2_ | POS | [M+H]^+^ | 6.24 | 111.0438 | -2.188 | 0.17 | - | 6225 |
| 181 | Pyroglutamic acid | C_5_H_7_NO_3_ | POS | [M+H]^+^, [M+NH_4_]^+^ | 1.3 | 130.0498 | -0.939 | -0.05 | - | 26424 |
| 182 | Pyromucic acid | C_5_H_4_O_3_ | POS | [M+H]^+^ | 1.4 | 113.0233 | -0.584 | - | - | 1370 |
|  |  |  | NEG | [M-H]^-^ | 2.2 | 111.0088 | 0.336 | - | - | 43678 |
|  |  |  |  | [M+H_2_O-H]^-^ | 2.31 | 129.0193 | -0.386 | - | - | 14902 |
| 183 | Pyrrolidonecarboxylic acid | C_5_H_7_NO_3_ | POS | [M+H]^+^ | 2.22 | 130.0497 | -1.636 | - | - | 17884 |
|  |  |  |  | [M+Na^+^ | 2.3 | 152.0316 | -1.194 | - | - | 1202 |
|  |  |  | NEG | [M-H]^-^ | 2.27 | 128.0353 | 0.081 | - | - | 7753 |
| 184 | Pyruvaldehyde | C_3_H_4_O_2_ | POS | [M+H]^+^ | 2.45 | 73.02835 | -0.765 | - | - | 1529 |
|  |  |  |  | [M+NH_4_]^+^ | 5.34 | 90.05486 | -1.076 | - | - | 1953 |
|  |  |  | NEG | [M+HCOO]^-^ | 1.37 | 117.0193 | 0.021 | - | - | 5357 |
| 185 | Quercetin | C_15_H_10_O_7_ | POS | [M+H]^+^ | 9.13 | 303.0498 | -0.667 | 0.01 | - | 1405 |
| 186 | Quinic acid | C_7_H_12_O_6_ | POS | [M+Na]^+^ | 1.16 | 215.0525 | -0.535 | 0.04 | - | 19184 |
|  |  |  |  | [M+K]^+^ | 1.16 | 231.0264 | -0.73 | 0.03 | - | 21650 |
|  |  |  |  | [M+H]^+^ | 1.39 | 193.0705 | -0.704 | 0.26 | - | 15973 |
|  |  |  |  | [M+NH_4_]^+^ | 1.41 | 210.097 | -1.159 | 0.29 | - | 994 |
|  |  |  | NEG | [M-H]^-^,  [2M-H]^-^ | 1.13 | 191.0563 | 1.024 | 0 | - | 88965 |
|  |  |  |  | [M+HCOO]^-^ | 1.17 | 237.0618 | 0.695 | 0.04 | - | 1987 |
| 187 | Quinolin-5-ol | C_9_H_7_NO | POS | [M+H]^+^ | 5.98 | 146.0597 | -2.455 | - | 946.6 | 46558 |
| 188 | Quinone | C_6_H_4_O_2_ | POS | [M+H]^+^ | 1.33 | 109.0283 | -1.029 | 0.3 | - | 2895 |
| 189 | Raffinose | C_18_H_32_O_16_ | POS | [M+Na]^+^ | 1.26 | 527.158 | -0.559 | 0.08 | - | 3099 |
| 190 | Rhamnose | C_6_H_12_O_5_ | POS | [M+K]^+^ | 1.18 | 203.0313 | -1.448 | -0.02 | - | 1214 |
|  |  |  |  | [M+H]^+^ | 1.31 | 165.0756 | -1.082 | 0.11 | - | 1033 |
| 191 | Rutin | C_27_H_30_O_16_ | POS | [M+H]^+^ | 7.38 | 611.1592 | -2.605 | 0.21 | - | 2640 |
| 192 | Salicin | C_13_H_18_O_7_ | POS | [M+NH_4_]^+^ | 6.27 | 304.1385 | -0.921 | - | - | 4037 |
|  |  |  |  | [M+H]^+^ | 7.07 | 287.1121 | -1.686 | - | - | 1420 |
|  |  |  | NEG | [M+HCOO]^-^ | 5.4 | 331.103 | -1.508 | - | - | 1837 |
| 193 | Shikimic acid | C_7_H_10_O_5_ | POS | [M+H]^+^ | 1.37 | 175.0598 | -1.537 | 0.05 | - | 1598 |
| 194 | Succinic acid | C_4_H_6_O_4_ | POS | [M+Na]^+^ | 2.4 | 141.0156 | -1.503 | 0.1 | - | 6202 |
|  |  |  |  | [M+H-H_2_O]^+^, [M+H]^+^ | 2.42 | 101.0232 | -1.536 | 0.12 | - | 24818 |
|  |  |  | NEG | [M-H]^-^,  [M-H-H_2_O]^-^ | 2.55 | 117.0193 | -0.198 | 0.25 | - | 70579 |
| 195 | Succinic acid semialdehyde | C_4_H_6_O_3_ | POS | [M+NH_4_]^+^ | 5.68 | 120.0655 | -0.05 | - | - | 1338 |
|  |  |  | NEG | [M-H]^-^ | 1.85 | 101.0245 | 1.85 | - | - | 8960 |
|  |  |  |  | [M+HCOO]^-^ | 2.16 | 147.0299 | 2.16 | - | - | 4815 |
| 196 | Succinylacetone | C_7_H_10_O_4_ | POS | [M+NH_4_]^+^ | 2.62 | 176.0913 | -2.46 | - | - | 2596 |
|  |  |  |  | [M+H]^+^, [M+Na]^+^ | 8.45 | 159.0649 | -2.214 | - | - | 19562 |
|  |  |  | NEG | [M-H]^-^ | 6.95 | 157.0506 | -0.407 | - | - | 1666 |
| 197 | Symmetric dimethylarginine | C_8_H_18_N_4_O_2_ | POS | [M+H]^+^ | 1.17 | 203.1501 | -0.703 | 0.03 | - | 15176 |
| 198 | Syringic acid | C_9_H_10_O_5_ | POS | [M+NH_4_-CO_2_]^+^, [M+H+CH_3_CN]^+^ | 6.98 | 172.0965 | -1.815 | 0.26 | - | 7171 |
| 199 | Terephthalic acid | C_8_H_6_O_4_ | POS | [M+H-H_2_O]^+^, [M+H]^+^ | 19.14 | 149.0229 | -2.693 | 0.11 | - | 15563 |
| 200 | Tetrahydroharman-3-carboxylic acid | C_13_H_14_N_2_O_2_ | POS | [M+H]^+^ | 6.81 | 231.1126 | -1.095 | - | 879.2 | 43274 |
| 201 | Trans-Aconitic acid | C_6_H_6_O_6_ | POS | [M+Na]^+^ | 1.86 | 197.0055 | -0.828 | - | - | 11107 |
|  |  |  | NEG | [M-H]^-^ | 2.23 | 173.0091 | -0.12 | - | - | 28805 |
| 202 | Trans-Ferulic acid | C_10_H_10_O_4_ | POS | [M+H+CH_3_CN]^+^ | 7.47 | 236.0912 | -2.254 | -0.19 | - | 2930 |
| 203 | Traumatic acid | C_12_H_20_O_4_ | POS | [M+Na]^+^ | 10.38 | 251.1251 | -1.044 | 0.03 | - | 1764 |
|  |  |  | NEG | [M-H]^-^ | 10.38 | 227.1285 | -2.008 | 0.03 | - | 7774 |
| 204 | Trehalose | C_12_H_22_O_11_ | POS | [M+NH_4_]^+^, [M+H]^+^ | 1.4 | 360.1496 | -1.12 | 0.16 | - | 15675 |
| 205 | Tri(3-chloropropyl) phosphate | C_9_H_18_Cl_3_O_4_P | POS | [M+H]^+^, [M+Na]^+^ | 12.35 | 327.0075 | -2.33 | - | 992.4 | 16208 |
| 206 | Triethylphosphate | C_6_H_15_O_4_P | POS | [M+H]^+^, [M+K]^+^, [M+Na]^+^ | 8.77 | 183.0777 | -2.265 | - | 986 | 391964 |
| 207 | Trigonelline | C_7_H_7_NO_2_ | POS | [M+H]^+^ | 1.17 | 138.0548 | -1.075 | 0.04 | - | 11883 |
| 208 | Tyramine | C_8_H_11_NO | POS | [M+H]^+^ | 3.32 | 138.0911 | -2.117 | - | - | 1985 |
| 209 | Umbelliferone | C_9_H_6_O_3_ | POS | [M+H]^+^ | 5.77 | 163.0389 | -0.591 | - | - | 3367 |
|  |  |  |  | [M+H+CH_3_CN]^+^ | 6.14 | 204.0652 | -1.498 | - | - | 82713 |
|  |  |  |  | [M+K]^+^ | 21.23 | 200.9953 | 2.219 | - | - | 2872 |
| 210 | Uracil | C_4_H_4_N_2_O_2_ | POS | [M+H]^+^ | 2.23 | 113.0343 | -1.866 | - | - | 1436 |
| 211 | Ureidopropionic acid | C_4_H_8_N_2_O_3_ | POS | [M+Na]^+^ | 1.21 | 155.0426 | -0.697 | -0.14 | - | 3909 |
|  |  |  |  | [M+H]^+^ | 1.29 | 133.0606 | -1.01 | -0.06 | - | 36792 |
| 212 | Uridine | C_9_H_12_N_2_O_6_ | POS | [M+H]^+^ | 2.19 | 245.0768 | 0.068 | - | - | 1278 |
|  |  |  | NEG | [M-H]^-^ | 2.83 | 243.0621 | -0.871 | - | - | 1582 |
| 213 | Urocanic acid | C_6_H_6_N_2_O_2_ | POS | [M+NH_4_]^+^ | 1.16 | 156.0766 | -0.975 | -0.19 | - | 11740 |
| 214 | Ursodeoxycholic acid | C_24_H_40_O_4_ | POS | [M+Na]^+^ | 17.34 | 415.2813 | -2.197 | - | - | 1140 |
| 215 | Vanillylmandelic acid | C_9_H_10_O_5_ | POS | [M+H+CH_3_CN]^+^ | 2.18 | 240.0862 | -1.82 | - | - | 1564 |
|  |  |  |  | [M+NH_4_]^+^ | 6.09 | 216.0867 | 0.263 | - | - | 1169 |
| 216 | Vitexin 4-O-glucoside | C_27_H_30_O_15_ | POS | [M+H]^+^ | 7.28 | 595.1648 | -1.645 | - | 920.4 | 15222 |
| 217 | Xanthurenic acid | C_10_H_7_NO_4_ | POS | [M+H]^+^ | 5.61 | 206.0444 | -1.874 | -0.09 | - | 18307 |
|  |  |  | NEG | [M-H]^-^ | 5.61 | 204.0301 | -1.053 | -0.1 | - | 5058 |
| 218 | Naringenin-7-O-glucoside | C_21_H_22_O_10_ | POS | [M+H]^+^ | 6.36 | 435.128 | -1.278 | 0.11 | - | 9238 |
| 219 | Apigenin-7-O-glucoside (Apigetrin) | C_21_H_20_O_10_ | POS | [M+H]^+^ | 6.57 | 433.1127 | -0.52 | 0.1 | - | 1905 |
|  |  |  | NEG | [M+H_2_O-H]^-^ | 6.45 | 449.1082 | -1.657 | -0.02 | - | 13261 |
| 220 | 3,6,2',3'-Tetrahydroxyflavone | C_15_H_10_O_6_ | POS | [M+H]^+^ | 7.73 | 287.0545 | -1.71 | -0.19 | - | 19430 |
| 221 | Demethyl medicarpin | C_15_H_12_O_4_ | POS | [M+H]^+^ | 8.34 | 257.0804 | -1.537 | 0.11 | - | 15826 |
|  |  |  | NEG | [M+HCOO]^-^ | 8 | 301.0711 | -2.165 | -0.23 | - | 987 |
| 222 | Sorbifolin | C_16_H_12_O_6_ | POS | [M+H]^+^ | 8.77 | 301.0715 | 2.599 | -0.21 | - | 1534 |
|  |  |  |  | [M+H]^+^,  [M+H-H_2_O]^+^ | 9.16 | 301.0701 | -1.816 | 0.18 | - | 31040 |
| 223 | Baicalein | C_15_H_10_O_5_ | POS | [M+H]^+^ | 11.39 | 271.0594 | -2.428 | -0.04 | - | 1128 |
| 224 | Asiatic acid | C_30_H_48_O_5_ | POS | [M+NH_4_]^+^ | 17.51 | 506.3831 | -2.453 | -0.3 | - | 1542 |
| 225 | Pygenic acid C b | C_30_H_48_O_6_ | POS | [M+NH_4_]^+^ | 17.64 | 522.3781 | 0.381 | -0.1 | - | 1172 |
| 226 | (1R,3R,4S,5R)-1,3,4-trihydroxy-5-[(E)-3-(4-hydroxyphenyl)prop-2-enoyl]oxycyclohexane-1-carboxylic acid | C_16_H_18_O_8_ | NEG | [M-H]^-^ | 6.26 | 337.0926 | -0.663 | - | 987.7 | 103112 |
| 227 | (1S)-1,5-Anhydro-2-O-.alpha.-L-arabinopyranosyl-1-(5,7-dihydroxy-2-(4-hydroxyphenyl)-4-oxo-4H-chromen-6-yl)-D-glucitol | C_26_H_28_O_14_ | NEG | [M-H]^-^ | 7.27 | 563.1392 | -2.484 | - | 856.7 | 12569 |
| 228 | (3-(Hexopyranosyloxy)-2-((2Z)-pent-2-en-1-yl)cyclopentyl)acetic acid | C_18_H_30_O_8_ | NEG | [M-H]^-^ | 7.9 | 373.1861 | -1.931 | - | 961.2 | 10194 |
|  |  |  |  | [M-H]^-^, [M+HCOO]^-^ | 8.09 | 373.1862 | -1.619 | - | 968.2 | 15004 |
| 229 | (4-(β-D-Glucopyranosyloxy)phenyl)acetic acid | C_14_H_18_O_8_ | NEG | [M-H]^-^ | 6.45 | 313.0926 | -1.028 | - | 978.4 | 42601 |
| 230 | (9Z)-5,8,11-Trihydroxyoctadec-9-enoic acid | C_18_H_34_O_5_ | NEG | [M-H]^-^ | 10.38 | 329.2328 | -1.688 | - | 895.9 | 12217 |
| 231 | 1,11-Undecanedicarboxylic acid | C_13_H_24_O_4_ | NEG | [M-H]^-^ | 11.48 | 243.1597 | -2.098 | 0.13 | - | 2169 |
| 232 | 1-[8-(β-D-Glucopyranosyloxy)-1,6-dihydroxy-3-methyl-2-naphthalenyl]ethanone | C_19_H_22_O_9_ | NEG | [M-H]^-^ | 7.42 | 393.1185 | -1.541 | - | 968.9 | 10647 |
| 233 | 1-O-((2E,4E)-5-((1R,3S,5S,8S)-3,8-Dihydroxy-1,5-dimethyl-6-oxabicyclo[3.2.1]octan-8-yl)-3-methylpenta-2,4-dienoyl)-β-D-glucopyranose | C_21_H_32_O_10_ | NEG | [M-H]^-^ | 6.07 | 443.1916 | -1.418 | - | 813.7 | 4766 |
| 234 | 1-O-((2E,4E)-9-Carboxy-8-hydroxy-2,7-dimethylnona-2,4-dienoyl)-.beta.-D-glucopyranose | C_18_H_28_O_10_ | NEG | [M-H]^-^ | 6.94 | 403.1603 | -1.694 | - | 820.3 | 8769 |
| 235 | 1-Palmitoyl-2-hydroxy-sn-glycero-3-phospho-(1'-rac-glycerol) | C_22_H_45_O_9_P | NEG | [M-H]^-^ | 12.34 | 483.2715 | -2.815 | - | 959.8 | 6225 |
| 236 | 2,2-Dimethylsuccinic acid | C_6_H_10_O_4_ | NEG | [M+HCOO]^-^ | 6.34 | 191.0558 | -1.601 | 0.02 | - | 2785 |
| 237 | 2,3-Dihydroxbenzoic acid | C_7_H_6_O_4_ | NEG | [M+H_2_O-H]^-^ | 2.24 | 171.0298 | -0.76 |  | - | 1493 |
| 238 | 2-Isopropylmalic acid | C_7_H_12_O_5_ | NEG | [M-H]^-^ | 6.32 | 175.0612 | 0.211 | 0.12 | - | 1854 |
| 239 | 3,4-Dihydroxybenzaldehyde | C_7_H_6_O_3_ | NEG | [M-H]^-^ | 6.06 | 137.0243 | -0.753 | -0.05 | - | 2333 |
| 240 | 3,4-Dimethylbenzene-1-sulfonic acid | C_8_H_10_O_3_S | NEG | [M-H]^-^ | 6.85 | 185.0277 | -0.411 | - | 982.8 | 2631 |
| 241 | 3-(Benzoyloxy)-2-hydroxypropyl- β-D-glucopyranosiduronic acid | C_16_H_20_O_10_ | NEG | [M-H]^-^ | 7.19 | 371.0978 | -1.443 | - | 805.4 | 16623 |
| 242 | 3-(Cyclohexylamino)-2-hydroxy-1-propanesulfonic acid | C_9_H_19_NO_4_S | NEG | [M-H]^-^ | 8.98 | 236.0959 | -1.275 | - | 996.8 | 12382 |
| 243 | 3-Hydroxyisovaleric acid | C_5_H_10_O_3_ | NEG | [M-H]^-^ | 4.8 | 117.0558 | 0.711 | 0.11 | - | 985 |
| 244 | 3-O-Feruloylquinic acid | C_17_H_20_O_9_ | NEG | [M-H]^-^ | 7.11 | 367.103 | -1.337 | - | 871.5 | 12063 |
| 245 | 3-O-p-Coumaroylquinic acid | C_16_H_18_O_8_ | NEG | [M-H]^-^, [2M-H]^-^ | 7.22 | 337.0926 | -0.952 | - | 989.8 | 367099 |
| 246 | 4-(Hexopyranosyloxy)-3-methoxybenzoic acid | C_14_H_18_O_9_ | NEG | [M-H]^-^ | 7.05 | 329.0876 | -0.672 | - | 937.2 | 7988 |
| 247 | 4-Hydroxyisophthalic acid | C_8_H_6_O_5_ | NEG | [M-H]^-^ | 6.05 | 181.0142 | -0.325 | - | 977.1 | 12810 |
| 248 | 4-O-D-Glucopyranosyl-p-coumaric acid | C_15_H_18_O_8_ | NEG | [M+HCOO]^-^, [M-H]^-^ | 5.88 | 371.0978 | -1.874 | - | 987.2 | 25575 |
| 249 | 4-O-p-Coumaroylquinic acid | C_16_H_18_O_8_ | NEG | [M-H]^-^ | 6.76 | 337.0926 | -0.99 | - | 920.5 | 58373 |
| 250 | 5,7-dihydroxy-2-(4-hydroxyphenyl)-6-[3,4,5-trihydroxy-6-(hydroxymethyl)oxan-2-yl]chromen-4-one | C_21_H_20_O_10_ | NEG | [M-H]^-^ | 7.46 | 431.0977 | -1.547 | - | 897.5 | 30250 |
| 251 | 5-Hydroxytryptophol | C_10_H_11_NO_2_ | NEG | [M+HCOO]^-^ | 6.06 | 222.077 | -0.951 | -0.05 | - | 3243 |
| 252 | 6-Methoxyluteolin | C_16_H_12_O_7_ | NEG | [M-H]^-^ | 11.18 | 315.0503 | -2.63 | - | 806.2 | 1097 |
| 253 | Acetaminophen glucuronide | C_14_H_17_NO_8_ | NEG | [M+H_2_O-H]^-^ | 4.75 | 344.0989 | 0.495 | 0.12 | - | 1311 |
| 254 | Allose | C_6_H_12_O_6_ | NEG | [M+HCOO]^-^ | 1.06 | 225.0619 | 1.226 | -0.02 | - | 1521 |
|  |  |  |  | [M-H]^-^ | 1.09 | 179.0562 | 0.415 | 0.02 | - | 6324 |
| 255 | Aloesin | C_19_H_22_O_9_ | NEG | [M-H]^-^, [M+H_2_O-H]^-^, [2M-H]^-^ | 6.19 | 393.1188 | -0.915 | - | 971.6 | 876435 |
|  |  |  |  | [M-H]^-^ | 7.14 | 393.1184 | -1.844 | - | 972.2 | 12600 |
| 256 | Aloin | C_21_H_22_O_9_ | NEG | [M-H]^-^, [M+HCOO]^-^ | 8.34 | 417.1187 | -1.089 | - | 981.4 | 572039 |
| 257 | Alpha-hydroxyhippuric acid | C_9_H_9_NO_4_ | NEG | [M+H_2_O-H]^-^ | 5.84 | 212.057 | 2.546 | 0.22 | - | 881 |
| 258 | Benzamide | C_7_H_7_NO | NEG | [M+H_2_O-H]^-^ | 6.32 | 138.0561 | 0.252 | 0.02 | - | 1897 |
| 259 | Cis-Melilotoside | C_15_H_18_O_8_ | NEG | [M-H]^-^,  [2M-H]^-^, [M+HCOO]^-^ | 6.4 | 325.0927 | -0.799 | - | 991.9 | 139764 |
| 260 | D-Arginine | C_6_H_14_N_4_O_2_ | NEG | [M-H]^-^ | 1.06 | 173.1046 | 1.355 | 0.12 | - | 12847 |
| 261 | D-Maltose | C_12_H_22_O_11_ | NEG | [M+HCOO]^-^, [2M+Cl]^-^,  [M-H]^-^, [2M-H]^-^ | 1.14 | 387.1144 | -0.132 | 0.02 | - | 19363 |
| 262 | Daidzein | C_15_H_10_O_4_ | NEG | [M+HCOO]^-^ | 9.15 | 299.0558 | -1.11 | 0.3 | - | 66731 |
| 263 | Endocrocin | C_16_H_10_O_7_ | NEG | [M-H]^-^ | 9.55 | 313.0349 | -1.562 | - | 889.2 | 4835 |
| 264 | Gentisic acid 5-O-glucoside | C_13_H_16_O_9_ | NEG | [M-H]^-^ | 5.32 | 315.0718 | -1.24 | - | 987.3 | 13360 |
| 265 | Gluconic acid | C_6_H_12_O_7_ | NEG | [M-H]^-^ | 1.22 | 195.0511 | 0.215 | 0.13 | - | 758 |
| 266 | Hemiphloin | C_21_H_22_O_10_ | NEG | [M-H]^-^ | 7.32 | 433.1134 | -1.48 | - | 865.7 | 216418 |
| 267 | Homogentisic acid | C_8_H_8_O_4_ | NEG | [M-H]^-^ | 5.07 | 167.0347 | -1.621 | 0.15 | - | 1074 |
| 268 | Homoorientin | C_21_H_20_O_11_ | NEG | [M-H]^-^ | 7.03 | 447.0926 | -1.659 | - | 806.7 | 5435 |
| 269 | Hydroxyisocaproic acid | C_6_H_12_O_3_ | NEG | [M-H]^-^ | 7.08 | 131.0715 | 0.988 | 0 | - | 1680 |
| 270 | Hydroxyphenyllactic acid | C_9_H_10_O_4_ | NEG | [M-H]^-^ | 5.96 | 181.0504 | -1.374 | 0.14 | - | 1443 |
| 271 | Jasmonic acid | C_12_H_18_O_3_ | NEG | [M-H]^-^ | 10.16 | 209.118 | -1.603 | - | 814.1 | 1679 |
| 272 | Kaempferol-3-O-Glucoside | C_21_H_20_O_11_ | NEG | [M-H]^-^ | 7.95 | 447.0926 | -1.551 | - | 925.1 | 32901 |
| 273 | L-Histidine | C_6_H_9_N_3_O_2_ | NEG | [M-H]^-^ | 1.03 | 154.0622 | 0.233 | 0.11 | - | 2445 |
| 274 | L-Serine | C_3_H_7_NO_3_ | NEG | [M-H]^-^ | 1.05 | 104.0355 | 2.187 | 0.09 | - | 2129 |
| 275 | L-Threonine | C_4_H_9_NO_3_ | NEG | [M-H]^-^ | 1.09 | 118.051 | 0.604 | 0.08 | - | 3561 |
| 276 | L-Tyrosine | C_9_H_11_NO_3_ | NEG | [M-H]^-^ | 3 | 180.0665 | -0.422 | - | 807.8 | 46021 |
| 277 | Laurylsulfuric acid | C_12_H_26_O_4_S | NEG | [M-H]^-^ | 13.81 | 265.1473 | -2.371 | - | 967.3 | 22319 |
| 278 | Linoleic acid | C_18_H_32_O_2_ | NEG | [M+HCOO]^-^ | 14.88 | 325.2379 | -1.615 | - |  | 1386 |
| 279 | 9,12,15-Octadecatrienoic acid, 3-(hexopyranosyloxy)-2-hydroxypropyl ester, (9Z,12Z,15Z)- | C_27_H_46_O_9_ | NEG | [M+HCOO]^-^,  [M-H]^-^, [M+Cl]^-^ | 13.96 | 559.3106 | -2.75 | - | 976.1 | 97907 |
| 280 | Ornithine | C_5_H_12_N_2_O_2_ | NEG | [M-H]^-^ | 1.07 | 131.0826 | 0.264 | 0.15 | - | 1489 |
| 281 | Paracetamol sulfate | C_8_H_9_NO_5_S | NEG | [M-H]^-^ | 5.33 | 230.0128 | -0.133 | - | - | 18702 |
| 282 | Phosphoric acid | H_3_O_4_P | NEG | [M-H]^-^ | 1.01 | 96.96977 | 1.604 | -0.08 | - | 5559 |
| 283 | Ribonolactone | C_5_H_8_O_5_ | NEG | [M+H_2_O-H]^-^ | 1.11 | 165.0405 | 0.171 | -0.05 | - | 2482 |
| 284 | Salicylic acid | C_7_H_6_O_3_ | NEG | [M-H]^-^ | 8.85 | 137.0242 | -1.341 | 0.14 | - | 1225 |
| 285 | Saponarin | C_27_H_30_O_15_ | NEG | [M-H]^-^, [2M-H]^-^ | 6.92 | 593.1503 | -1.511 | - | 967 | 261401 |
| 286 | Sebacic acid | C_10_H_18_O_4_ | NEG | [M-H]^-^ | 9.22 | 201.1132 | -0.29 | 0.13 | - | 2011 |
| 287 | Sulfite | O_3_S | NEG | [M+H_2_O-H]^-^ | 21.62 | 96.96003 | -0.752 | - | - | 3457 |
| 288 | Threonic acid | C_4_H_8_O_5_ | NEG | [M-H]^-^ | 1.13 | 135.0297 | -1.538 | 0.02 | - | 6835 |
| 289 | Tiglylglycine | C_7_H_11_NO_3_ | NEG | [M+HCOO]^-^ | 5.92 | 202.0719 | -1.108 | 0.1 | - | 1516 |
| 290 | Undecanedioic acid | C_11_H_20_O_4_ | NEG | [M-H]^-^ | 10.02 | 215.1285 | -1.779 | 0.14 | - | 2095 |
| 291 | Luteolin 7-O-glucoside (Cynaroside) | C_21_H_20_O_11_ | NEG | [M+H_2_O-H]^-^ | 5.59 | 465.1032 | -1.381 | 0.18 | - | 39293 |
| 292 | 3',4'-Dihydroxyflavone | C_15_H_10_O_4_ | NEG | [M+HCOO]^-^ | 9.15 | 299.0558 | -1.11 | -0.07 | - | 66731 |
| 293 | 4'-Hydroxyflavone | C_15_H_10_O_3_ | NEG | [M+H_2_O-H]^-^ | 10.77 | 255.0659 | -1.259 | -0.13 | - | 2463 |
| 294 | Luteolin-4'-O-glucoside | C_21_H_20_O_11_ | NEG | [M+H_2_O-H]^-^ | 6.23 | 465.1034 | -0.851 | -0.03 | - | 17165 |
| 295 | 3,6,2',4'-Tetrahydroxyflavone | C_15_H_10_O_6_ | NEG | [M-H]^-^ | 6.23 | 285.0407 | 0.678 | -0.18 | - | 1446 |
| 296 | Isorhoifolin | C_27_H_30_O_14_ | NEG | [M+H_2_O-H]^-^ | 6.36 | 595.1657 | -2.126 | -0.05 | - | 10072 |
| 297 | Apiin | C_26_H_28_O_14_ | NEG | [M+HCOO]^-^ | 6.56 | 609.1449 | -1.993 | 0.1 | - | 22626 |
| 298 | 3,6,3',4'-Tetrahydroxyflavone | C_15_H_10_O_6_ | NEG | [M+H_2_O-H]^-^ | 6.61 | 303.0509 | -0.557 | -0.17 | - | 1327 |
| 399 | Apigenin-7-neohesperidoside (Rhoifolin) | C_27_H_30_O_14_ | NEG | [M+HCOO]^-^ | 7.02 | 623.1605 | -1.956 | 0.28 | - | 16357 |
| 300 | Formononetin-7-O-glucoside (Ononin) | C_22_H_22_O_9_ | NEG | [M+HCOO]^-^ | 8.02 | 475.1238 | -1.594 | -0.13 | - | 7663 |
| 301 | 6,4'-Dimethoxyisoflavone-7-glucoside (Wistin) | C_23_H_24_O_10_ | NEG | [M+HCOO]^-^ | 8.31 | 505.134 | -1.589 | -0.12 | - | 8231 |
|  |  |  |  | [M-H]^-^ | 8.58 | 459.129 | -1.793 | 0.15 | - | 99305 |
| 302 | Oct-1-en-3-yl Ara (1-6)Glu (NMR) | C_19_H_34_O_10_ | NEG | [M-H]^-^ | 9.04 | 421.207 | 0.214 | 0.3 | - | 968 |

^a^Data is provided for protonated ions.

**Table S2.** Metabolites localized in *Aloe vera* samples by both MSI methods used in the study.

| **Name** | **Molecular formula** | **^109^AgNPs-LDI** | | **LARAPPI/CI** | | **Figure** |
| --- | --- | --- | --- | --- | --- | --- |
|  |  | **Detected ion** | ***m/z*^a^** | **Detected ion** | ***m/z*^a^** |  |
| 3-Coumaric Acid^b, d, e^ | C_9_H_8_O_3_ | [M + ^109^Ag]^+^ | 272.9516 | [M - H]^-^ | 163.0398 | 1E |
| Ferulic acid^b, c^ | C_10_H_10_O_4_ | [M + K]^+^ | 233.0211 | [M - H]^-^ | 193.0506 | 1F |
| Gluconic acid^b, c^ | C_6_H_12_O_7_ | [M + H]^+^ | 197.0656 | [M - H]^-^ | 195.0511 | 1G |
| Kaempferol^b,c^ | C_15_H_10_O_6_ | [M + H]^+^ | 287.0550 | [M - H]^-^ | 285.0404 | 1H |
| 6-Phosphogluconic acid^b, c^ | C_6_H_13_O_10_P | [M + H]^+^ | 277.0319 | [M - H]^-^ | 275.0173 | 1I |
| Ribose 5-phosphate^b, c, d^ | C_5_H_11_O_8_P | [M + H]^+^ | 231.0264 | [M - H]^-^ | 229.0119 | 1J |
|  |  |  |  |  |  |  |
| Shikimic acid^b, c^ | C_7_H_10_O_5_ | [M + Na]^+^ | 197.0420 | [M - H]^-^ | 173.0453 | 1K |
| Sorbifolin^b,c^ | C1_6_H_12_O_6_ | [M + H]^+^ | 301.0706 | [M - H]^-^ | 299.0569 | 1L |
| 2-Allyl-1,4-dimethoxy-3-methyl-benzene^b^ | C_12_H_16_O_2_ | [M + ^109^Ag]^+^ | 301.0190 | [M - H]^-^ | 191.1077 | 2A |
| Aloenin aglycone^b^ | C_13_H_12_O_5_ | [M + K]^+^ | 287.0316 | [M - H]^-^ | 247.0612 | 2B |
| Apigenin-7-glyvuronyl^b^ | C_21_H_18_O_11_ | [M + K]^+^ | 485.0481 | [M - H]^-^ | 445.0776 | 2C |
| Arabinose^b^ | C_5_H_10_O_5_ | [M + ^109^Ag]^+^ | 258.9570 | [M - H]^-^ | 149.0455 | 2D |
| 3-Caffeoyl-5-coumaroylquinic acid^b^ | C_25_H_24_O_11_ | [M + ^109^Ag]^+^ | 609.0361 | [M - H]^-^ | 499.1246 | 2E |
| Chrysoerion-7-glucoside^b^ | C_22_H_22_O_11_ | [M + Na]^+^ | 485.1054 | [M - H]^-^ | 461.1089 | 2F |
| Ethyl citrate^b^ | C_8_H_12_O_7_ | [M + K]^+^ | 259.0215 | [M - H]^-^ | 219.0510 | 2G |
| 8-Glucosyl-7-methyl aloesol | C_20_H_26_O_9_ | [M + Na]^+^ | 433.1469 | [M - H]^-^ | 409.1504 | 2H |
| 2-Heptanol^b^ | C_7_H_16_O | [M + ^109^Ag]^+^ | 225.0243 | [M - H]^-^ | 115.1128 | 2A |
| Indole-3-acetic acid^b^ | C_10_H_9_NO_2_ | [M + K]^+^ | 214.0265 | [M - H]^-^ | 174.0560 | 2B |
| Isoeugentin^b^ | C_12_H_12_O_4_ | [M + K]^+^ | 259.0367 | [M - H]^-^ | 219.0663 | 2C |
| Malonyl-3,4-dicaffeooylquinic acid^b^ | C_28_H_26_O_15_ | [M + ^109^Ag]^+^ | 711.0313 | [M - H]^-^ | 601.1199 | 3D |
| Naringenin^b^ | C_15_H_12_O_5_ | [M + H]^+^ | 273.0758 | [M - H]^-^ | 271.0612 | 3E |
| Tartaric acid^b^ | C_4_H_6_O_6_ | [M + ^109^Ag]^+^ | 258.9206 | [M - H]^-^ | 149.0092 | 3F |
| Tetradecyne^b^ | C_14_H_26_ | [M + K]^+^ | 233.1666 | [M - H]^-^ | 193.1962 | 3G |
| Theaspirine^b^ | C_13_H_22_O | [M + K]^+^ | 233.1302 | [M - H]^-^ | 193.1598 | 3H |

^a^ Experimental monoisotopic mass of ion; ^b^ the metabolites identified by high precursor mass accuracy; ^c^ the metabolites identified by matching retention time; ^d^ the metabolites identified by matching isotopic pattern; ^e^ the metabolites identified by matching MS/MS fragment spectra; *m/z*: mass-to-charge ratio.

**
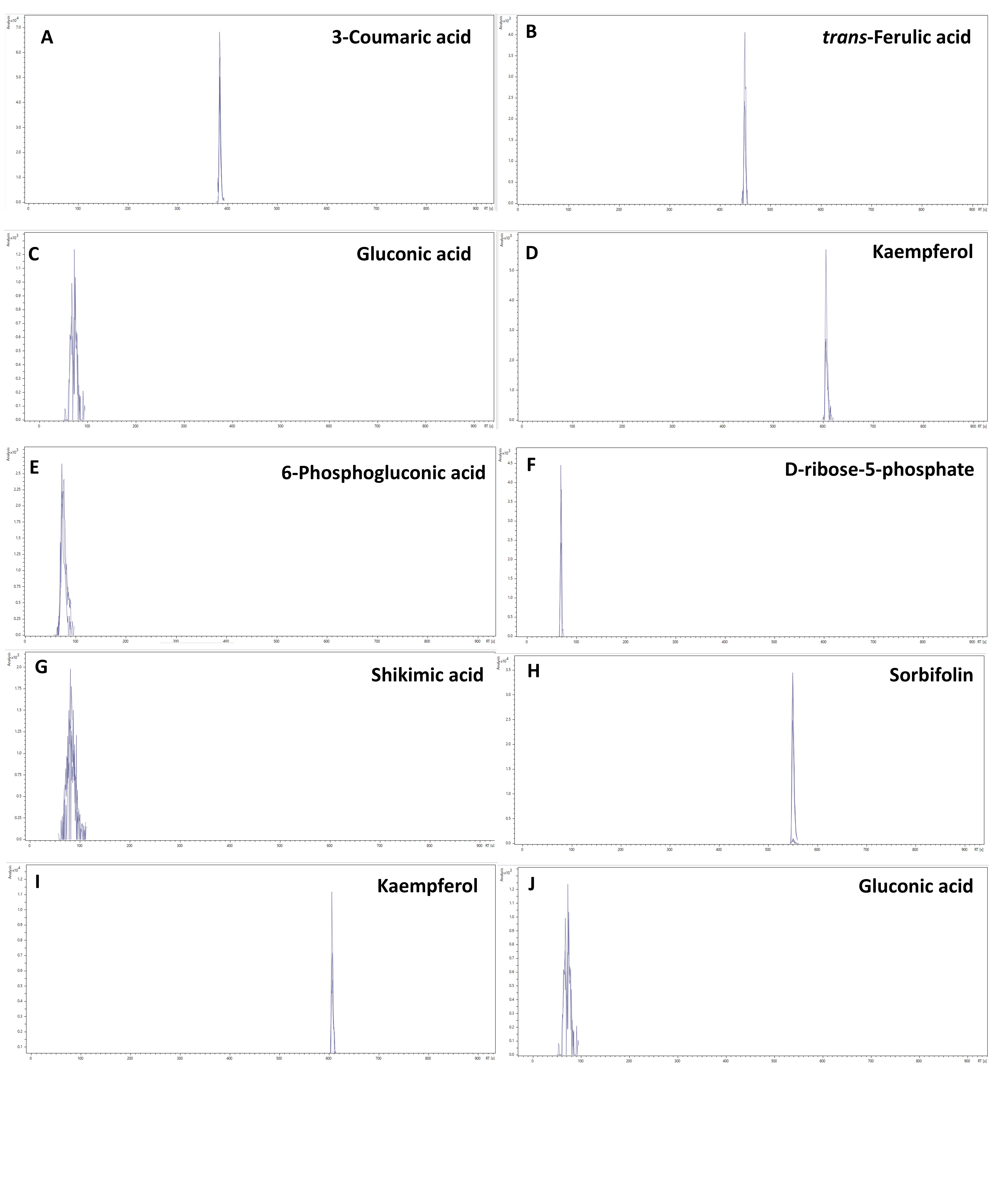
**

**Fig. S11** The UHPLC-HRMS chromatograms for selected compounds: A – 3-Coumaric acid (positive mode), B – trans-Ferulic acid (positive mode), C – Gluconic acid (positive mode), D – Kaempferol (positive mode), E – 6-Phosphogluconic acid (positive mode), F – D-ribose-5-phosphate (positive mode), G – Shikimic acid (positive mode), and H – Sorbifolin (positive mode), I – Kaempferol (negative mode), J - Gluconic acid (negative mode).

**Table S3.** Pathway analysis results.

| **Pathway** | **Total** | **Hits** | **Raw p** | **-log10(p)** | **Holm adjust** | **FDR** | **Impact** |
| --- | --- | --- | --- | --- | --- | --- | --- |
| Flavonoid biosynthesis | 53 | 31 | 2.34e-24 | 23.6310 | 2.13e-22 | 2.13e-22 | 0.4992 |
| Pentose phosphate pathway | 19 | 14 | 2.61e-13 | 12.5840 | 2.35e-11 | 1.19e-11 | 0.8240 |
| Phenylpropanoid biosynthesis | 35 | 16 | 1.50e-10 | 9.8245 | 1.33e-08 | 4.54e-09 | 0.4149 |
| Carbon fixation in photosynthetic organisms | 21 | 10 | 3.52e-07 | 6.4534 | 3.10e-05 | 8.01e-06 | 0.2438 |
| Phenylalanine, tyrosine and tryptophan biosynthesis | 22 | 9 | 6.83e-06 | 5.1654 | 0.00059 | 0.00012 | 0.3378 |
| Flavone and flavonol biosynthesis | 13 | 5 | 0.00127 | 2.8950 | 0.10952 | 0.01931 | 0.3333 |
| Alanine, aspartate and glutamate metabolism | 22 | 6 | 0.00308 | 2.5112 | 0.26194 | 0.04006 | 0.3166 |
| Arginine biosynthesis | 18 | 5 | 0.00638 | 2.1951 | 0.53608 | 0.07259 | 0.1709 |
| Citrate cycle (TCA cycle) | 20 | 5 | 0.01031 | 1.9866 | 0.85606 | 0.10429 | 0.2891 |
| Tyrosine metabolism | 18 | 4 | 0.03275 | 1.4848 | 1.00000 | 0.29799 | 0.2378 |
| Phenylalanine metabolism | 12 | 3 | 0.04658 | 1.3318 | 1 | 0.38533 | 0.7692 |
| Anthocyanin biosynthesis | 6 | 2 | 0.06093 | 1.2151 | 1 | 0.46208 | 0 |
| Isoflavonoid biosynthesis | 14 | 3 | 0.06960 | 1.1574 | 1 | 0.47071 | 0 |
| Pyruvate metabolism | 23 | 4 | 0.07242 | 1.1402 | 1 | 0.47071 | 0.1575 |
| D-Amino acid metabolism | 7 | 2 | 0.08147 | 1.0890 | 1 | 0.49425 | 0 |
| Monobactam biosynthesis | 8 | 2 | 0.10377 | 0.9839 | 1 | 0.53523 | 0 |
| Glycolysis / Gluconeogenesis | 26 | 4 | 0.10422 | 0.9821 | 1 | 0.53523 | 0.2542 |
| Butanoate metabolism | 17 | 3 | 0.11175 | 0.9518 | 1 | 0.53523 | 0.2273 |
| Pentose and glucuronate interconversions | 17 | 3 | 0.11175 | 0.9518 | 1 | 0.53523 | 0.3333 |
| Tropane, piperidine and pyridine alkaloid biosynthesis | 9 | 2 | 0.12749 | 0.8945 | 1 | 0.58008 | 0 |
| Glyoxylate and dicarboxylate metabolism | 29 | 4 | 0.14133 | 0.8498 | 1 | 0.6048 | 0.0917 |
| Caffeine metabolism | 10 | 2 | 0.15232 | 0.8172 | 1 | 0.6048 | 0 |
| Amino sugar and nucleotide sugar metabolism | 52 | 6 | 0.15286 | 0.8157 | 1 | 0.6048 | 0.2856 |
| Starch and sucrose metabolism | 22 | 3 | 0.19744 | 0.7046 | 1 | 0.6916 | 0.0924 |
| Ascorbate and aldarate metabolism | 22 | 3 | 0.19744 | 0.7046 | 1 | 0.6916 | 0.2245 |
| Glycine, serine and threonine metabolism | 33 | 4 | 0.19760 | 0.7042 | 1 | 0.6916 | 0.1782 |
| Tryptophan metabolism | 25 | 3 | 0.25471 | 0.5940 | 1 | 0.85848 | 0.5862 |
| C5-Branched dibasic acid metabolism | 6 | 1 | 0.35475 | 0.4501 | 1 | 1 | 0 |
| Isoquinoline alkaloid biosynthesis | 6 | 1 | 0.35475 | 0.4501 | 1 | 1 | 0.4118 |
| Fructose and mannose metabolism | 18 | 2 | 0.36468 | 0.4381 | 1 | 1 | 0.3780 |
| Cysteine and methionine metabolism | 46 | 4 | 0.40739 | 0.3900 | 1 | 1 | 0.0526 |
| Valine, leucine and isoleucine biosynthesis | 22 | 2 | 0.46542 | 0.3322 | 1 | 1 | 0.1645 |
| Lysine biosynthesis | 9 | 1 | 0.48206 | 0.3169 | 1 | 1 | 0 |
| Lipoic acid metabolism | 24 | 2 | 0.51224 | 0.2905 | 1 | 1 | 0 |
| Sesquiterpenoid and triterpenoid biosynthesis | 11 | 1 | 0.55276 | 0.2575 | 1 | 1 | 0.2419 |
| Biosynthesis of various plant secondary metabolites | 26 | 2 | 0.55632 | 0.2547 | 1 | 1 | 0 |
| Glutathione metabolism | 27 | 2 | 0.57729 | 0.2386 | 1 | 1 | 0 |
| Galactose metabolism | 27 | 2 | 0.57729 | 0.2386 | 1 | 1 | 0.0562 |
| Vitamin B6 metabolism | 12 | 1 | 0.58444 | 0.2333 | 1 | 1 | 0 |
| Arginine and proline metabolism | 28 | 2 | 0.59755 | 0.2236 | 1 | 1 | 0.1254 |
| Purine metabolism | 74 | 5 | 0.60675 | 0.2170 | 1 | 1 | 0.0413 |
| Riboflavin metabolism | 13 | 1 | 0.61390 | 0.2119 | 1 | 1 | 0 |
| Nicotinate and nicotinamide metabolism | 13 | 1 | 0.61390 | 0.2119 | 1 | 1 | 0 |
| Cyanoamino acid metabolism | 31 | 2 | 0.65396 | 0.1844 | 1 | 1 | 0 |
| Ubiquinone and other terpenoid-quinone biosynthesis | 47 | 3 | 0.65510 | 0.1837 | 1 | 1 | 0.0010 |
| Sulfur metabolism | 15 | 1 | 0.66675 | 0.1760 | 1 | 1 | 0 |
| Folate biosynthesis | 33 | 2 | 0.68799 | 0.1624 | 1 | 1 | 0.0262 |
| Histidine metabolism | 16 | 1 | 0.69042 | 0.1609 | 1 | 1 | 0 |
| beta-Alanine metabolism | 18 | 1 | 0.73288 | 0.1350 | 1 | 1 | 0 |
| Thiamine metabolism | 22 | 1 | 0.80125 | 0.0962 | 1 | 1 | 0 |
| Pantothenate and CoA biosynthesis | 25 | 1 | 0.84087 | 0.0753 | 1 | 1 | 0 |
| Sphingolipid metabolism | 27 | 1 | 0.86283 | 0.0641 | 1 | 1 | 0 |
| Inositol phosphate metabolism | 28 | 1 | 0.87266 | 0.0592 | 1 | 1 | 0 |
| alpha-Linolenic acid metabolism | 28 | 1 | 0.87266 | 0.0592 | 1 | 1 | 0 |
| Terpenoid backbone biosynthesis | 31 | 1 | 0.89814 | 0.0467 | 1 | 1 | 0 |
| Fatty acid degradation | 37 | 1 | 0.93493 | 0.0292 | 1 | 1 | 0 |
| Glycerophospholipid metabolism | 38 | 1 | 0.93962 | 0.0270 | 1 | 1 | 0 |
| Pyrimidine metabolism | 41 | 1 | 0.95178 | 0.0215 | 1 | 1 | 0 |
| Steroid biosynthesis | 44 | 1 | 0.96151 | 0.0170 | 1 | 1 | 0.0374 |
